# Mixotrophy in a Local Strain of *Nannochloropsis* *granulata* for Renewable High-Value Biomass Production on the West Coast of Sweden

**DOI:** 10.3390/md20070424

**Published:** 2022-06-28

**Authors:** Valeria Villanova, Christian Galasso, Giovanni Andrea Vitale, Gerardo Della Sala, Johan Engelbrektsson, Niklas Strömberg, Kashif Mohd Shaikh, Mats X. Andersson, Fortunato Palma Esposito, Susanne Ekendahl, Donatella De Pascale, Cornelia Spetea

**Affiliations:** 1 Department of Biological and Environmental Sciences, University of Gothenburg, 405 30 Gothenburg, Sweden; mohd.kashif.shaikh@bioenv.gu.se (K.M.S.); mats.andersson@bioenv.gu.se (M.X.A.); 2 Department of Ecosustainable Marine Biotechnology, Stazione Zoologica Anton Dohrn, Calabria Marine Centre, C. da Torre Spaccata, 87071 Amendolara, Italy; christian.galasso@szn.it; 3 Department of Ecosustainable Marine Biotechnology, Stazione Zoologica Anton Dohrn, Via A.F. Acton, Molosiglio, 80133 Napoli, Italy; giovanniandrea.vitale@szn.it (G.A.V.); gerardo.dellasala@szn.it (G.D.S.); fortunato.palmaesposito@szn.it (F.P.E.); donatella.depascale@szn.it (D.D.P.); 4 Department of Chemistry, Biomaterials and Textiles, RISE Research Institutes of Sweden AB, 471 56 Gothenburg, Sweden; johan@addscience.se (J.E.); niklas@stromtech.se (N.S.); miljosus@miljosus.se (S.E.)

**Keywords:** *Nannochloropsis*, mixotrophy, photobioreactors, CHN analysis, carotenoids, polyunsaturated fatty acids, metabolomics, bioassay, cell death pathway, autophagy, antitumoral activity

## Abstract

A local strain of *Nannochloropsis granulata* (*Ng*) has been reported as the most productive microalgal strain in terms of both biomass yield and lipid content when cultivated in photobioreactors that simulate the light and temperature conditions during the summer on the west coast of Sweden. To further increase the biomass and the biotechnological potential of this strain in these conditions, mixotrophic growth (i.e., the simultaneous use of photosynthesis and respiration) with glycerol as an external carbon source was investigated in this study and compared with phototrophic growth that made use of air enriched with 1–2% CO_2_. The addition of either glycerol or CO_2_-enriched air stimulated the growth of *Ng* and theproduction of high-value long-chain polyunsaturated fatty acids (EPA) as well as the carotenoid canthaxanthin. Bioassays in human prostate cell lines indicated the highest antitumoral activity for *Ng* extracts and fractions from mixotrophic conditions. Metabolomics detected betaine lipids specifically in the bioactive fractions, suggesting their involvement in the observed antitumoral effect. Genes related to autophagy were found to be upregulated by the most bioactive fraction, suggesting a possible therapeutic target against prostate cancer progression. Taken together, our results suggest that the local *Ng* strain can be cultivated mixotrophically in summer conditions on the west coast of Sweden for the production of high-value biomass containing antiproliferative compounds, carotenoids, and EPA.

## 1. Introduction

Microalgae are unicellular photosynthetic microorganisms that originated from endosymbiotic events in which a heterotrophic ancestor fused with various photoautotrophic (photosynthetic) organisms [1]. Thanks to this evolutionary history, they possess both photosynthetic and respiratory organelles (chloroplasts and mitochondria, respectively) and hence exhibit trophic flexibility. Although most microalgae are photoautotrophs, some of them are also able to use organic carbon via respiration, either in the dark (heterotrophs) or in a light-dependent manner (mixotrophs). Mixotrophy is the trophic mode in which both CO_2_ and organic carbon are assimilated simultaneously, owing to the activation of both respiration and photosynthesis. It can be employed as a method to increase the productivity of microalgae that are cultivated in low light conditions. To minimize the additional cost of organic carbon supplementation, industrial wastewater and biodiesel waste (i.e., glycerol) are often used for algae cultivation and biomass production (as reviewed by Villanova et al. 2021 [2]). The Eustigmatophyceae *Nannochloropsis* genus has been extensively used for biotechnological applications such as the production of biofuel [3,4] and fish feed [5]. Moreover, the *Nannochloropsis* genus is a well-known source of polyunsaturated fatty acids, such as eicosapentaenoic acid (EPA) and carotenoids, that are important for human health [6,7]. Finally, in vitro assays have shown interesting anti-cancer activity of the *Nannochloropsis* biomass on different human cell lines, opening new perspectives for the nutraceutical application of this genus [8,9,10].

Mixotrophic growth of the *Nannochloropsis* genus (i.e., *Nannochloropsis gaditana* and *Nannochloropsis salina*) been investigated using glucose, glycerol, and acetate as external organic carbon sources or by adding the bacteria-rich medium Lysogeny broth (LB) [11,12,13,14]. However, only a handful of research projects have focused on understanding the industrial potential of growing *Nannochloropsis* under this growth mode [12,14].

There is an increasing need for sustainable resources for the production of food, feed, oil-based materials, and energy. Microalgae have a great potential in this respect and can be cultivated outdoors in conditions resembling those in natural habitats. However, to ensure high productivity, it is necessary to exploit the natural diversity of microalgae and select local species and strains that can adjust their physiology to changing environments. This is particularly important in the Nordic countries, which have large variations in light intensity and temperature throughout the year [15]. The overall goal of this work was to employ mixotrophy as a strategy for maximizing the outdoor productivity of a *Nannochloropsis granulata* strain (*Ng*), isolated from the Skagerrak in the northeast Atlantic Ocean [16]. A previous study reported that the local *Ng* was the most productive strain (compared to 166 strains of *Skeletonema marinoi*), in terms of both biomass yield and lipid content when cultivated in photobioreactors that simulate the light and temperature conditions during the summer on the west coast of Sweden [17]. More specifically, in summer, *Ng* reached 3.5 g/L of biomass containing 40% lipids, i.e., 7 and 1.5-fold, respectively, more than the most productive *Skeletonema marinoi* strain in the same conditions [17]. Here, we aimed to further increase the biomass and lipid productivity of *Ng* under simulated summer conditions on the west coast of Sweden by the addition of glycerol, and also to expand the biotechnological potential of the biomass in terms of biofuel, food production, and antitumoral activity. To reach this aim, an interdisciplinary approach was applied for the first time on this strain in order to allow for in-depth physiological and biochemical characterization in mixotrophy as compared to phototrophy; this was combined with the production of high-value molecules and tested on a small scale (i.e., Multi-Cultivator) before being validated on a larger scale (i.e., photobioreactors).

## 2. Results

### 2.1. Effect of Mixotrophy on Growth Rates, Photosynthesis, and Biomass Yield in a Multi-Cultivator System

First, we tested the effect of various external carbon sources on *Ng* by monitoring the growth changes in phototrophic and mixotrophic cultures in a Multi-Cultivator MC 1000-OD (Photon System Instruments, Drásov, Czech Republic), with a volume of 80 mL optimised f/2 medium with air bubbling (as described in Section 4.1.1 and shown in Figure S1a). We used a constant light intensity of 300 µmol photons m^−2^ s^−1^ at a temperature of 20 °C, corresponding to the average light intensity and temperature, respectively, during the summer season in Gothenburg. *Ng* was able to grow in mixotrophy using either glycerol or glucose (but not acetate) as an external carbon source, therefore confirming previous results for *Nannochloropsis gaditana* [14] (see Supplementary Materials Figure S2). Since glycerol is a by-product of biofuel production, and hence a cheap carbon source, it was selected for further experiments with *Ng* in mixotrophic conditions [18].

**Figure 1:**
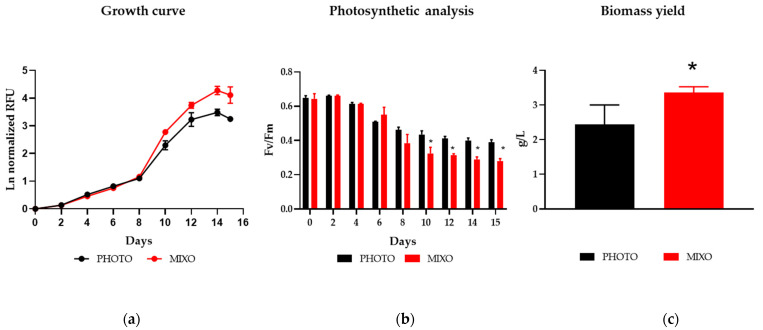
(**a**) Growth profile, (**b**) photosynthetic analysis, and (**c**) final biomass yield of *Nannochloropsis granulata* grown in a Multi-Cultivator at 20 °C, with constant light at 300 µmol photons m^−2^ s^−1^, under mixotrophy with 10 mM glycerol (MIXO) or under phototrophy with air (PHOTO). The growth was monitored in relative chlorophyll fluorescence units (RFU), and plotted data are values normalized at time zero. Data were considered significant for *p*-values < 0.05 (* *p* < 0.05). Each point is expressed as mean ± standard deviation (*n* = 3). Schemes follow the same formatting.

During the first days of cultivation (0–6 days) in the Multi-Cultivator, the cells grew similarly in mixotrophy and phototrophy. From day 10, the growth in mixotrophy was significantly enhanced as compared to phototrophy (Figure 1a).

In the first 4 days of the experiment, *Ng* displayed a maximum quantum efficiency of PSII photochemistry (i.e., F_v_/F_m_) of about 0.6 in both phototrophy and mixotrophy (Figure 1b), corresponding to a healthy physiological status in the genus [16]. From day 6, a decrease in photosynthetic performance was detected in all tested samples, but this decrease was significantly more pronounced in mixotrophy from day 10. The decrease in Fv/Fm during mixotrophic growth has been previously reported for *Nannochloropsis gaditana* and the diatom *Phaeodactylum tricornutum* [11,19]. The enhanced growth together with the decrease in photosynthesis from day 10 in mixotrophic samples indicate a switch from the phototrophic to the mixotrophic regime. Moreover, the samples grown in mixotrophy showed an increase of about 1.4-fold in the final biomass (Figure 1c). Taken together, these data showed that *Ng* was able to grow under mixotrophy using glycerol as an external carbon source in the tested conditions.

### 2.2. Effect of Mixotrophy on Growth Rate, Biomass Yield, Energy Production, and Nutrient Removal in Photobioreactors

**Figure 2:**
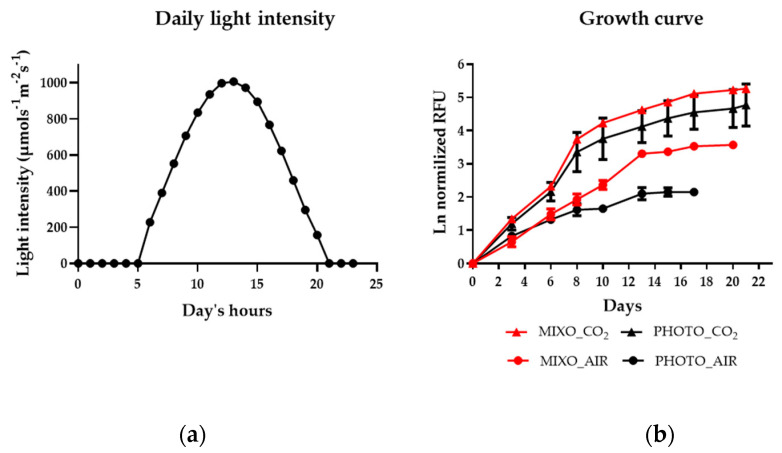
(**a**) Light cycle used in the environmental photobioreactors (ePBRs) for growing *Nannochloropsis granulata*, simulating the summer conditions in Gothenburg; (**b**) Growth profile of *Nannochloropsis granulata* grown with light in a medium injected with air (PHOTO_AIR, black circle); in a medium supplemented with 10 mM glycerol and injected with air (MIXO_AIR, red circle); in a medium injected with 1–2% CO_2_-enriched air (PHOTO_CO_2_, black square); in a medium supplemented with 10 mM glycerol and injected with 1–2% CO_2_-enriched air (MIXO_CO_2_, red square). The growth was monitored in relative chlorophyll fluorescence units (RFU), and plotted data are values normalized at time zero. Each time point is the mean ± standard deviation (*n* = 3–5).

Next, the cultivation was upscaled to environmental photobioreactors (ePBRs) of 1L (as described in Section 4.1.2 and shown in Figure S1b), using a constant temperature of 20 °C and the simulated photoperiod of summer in Gothenburg, Sweden (Figure 2a). In particular, the mixotrophic growth of samples injected only with air (i.e., 0.04% CO_2_) or with 1–2% CO_2_-enriched air (MIXO_AIR and MIXO_CO_2_, respectively) was compared with a phototrophic control grown with the same aeration system (PHOTO_AIR and PHOTO_CO_2_, respectively). When supplied with air in the ePBR, the addition of glycerol (MIXO_AIR) stimulated growth (expressed as relative fluorescence units, RFU) as compared to the phototrophic condition (PHOTO_AIR) starting from day 10 (Figure 2b), which is in line with the observations in the Multi-Cultivator (Figure 1a). Moreover, both samples supplied with CO_2_, i.e., MIXO_CO_2_ and PHOTO_CO_2_, further experienced stimulated growth from day 3 as compared to the samples PHOTO_AIR and MIXO_AIR (Figure 2b). There was no difference in the growth profiles of the conditions PHOTO_CO_2_ and MIXO_CO_2_; hence, the better growth in these conditions as compared to the others could be attributed to the addition of CO_2_ rather than glycerol [20].

To better compare the four different conditions, both growth (i.e., specific growth rate, biomass yield, and productivity) and energy parameters (calorific value and energetic productivity) were determined. There were no significant differences among PHOTO_CO_2_, MIXO_CO_2_, and MIXO_AIR in these parameters, and they were all significantly more performant than the condition PHOTO_AIR. More specifically, the specific growth rate, biomass yield, and productivity in these three conditions significantly increased by 1.6-, 3.6-, and 3.6-fold, respectively, when compared to PHOTO_AIR (Table 1). In the same conditions, the calorific value and the energetic productivity of the obtained biomass were enhanced 1.4- and 5-fold, respectively. This indicates that the addition of either/both organic or/and inorganic carbon similarly improved biomass and energy production in the tested conditions. Moreover, both nitrate and phosphate consumption were increased in MIXO_AIR as compared to PHOTO_AIR by about 4-fold (Table 1). In MIXO_CO_2_, only phosphate consumption was increased as compared to PHOTO_CO_2_, while nitrate consumption was similar in the three conditions MIXO_AIR, PHOTO_CO_2_, and MIXO_CO_2_. The glycerol consumption was similar among the mixotrophic samples. In addition to reduced growth and nutrient consumption, the samples grown in PHOTO_AIR showed the highest ash content (Table 1), about 3-fold higher than the other three conditions. The PHOTO_CO_2_ data for *Ng* are in good agreement with those reported previously for *Ng* in summer conditions [17]. Taken together, the new data show that the addition of glycerol improves the growth and biomass production of *Ng* in summer conditions, reaching productivities similar to those obtained in phototrophy with CO_2_.

### 2.3. Biomass Composition, Pigment, and Fatty Acid Profiles

**Figure 3:**
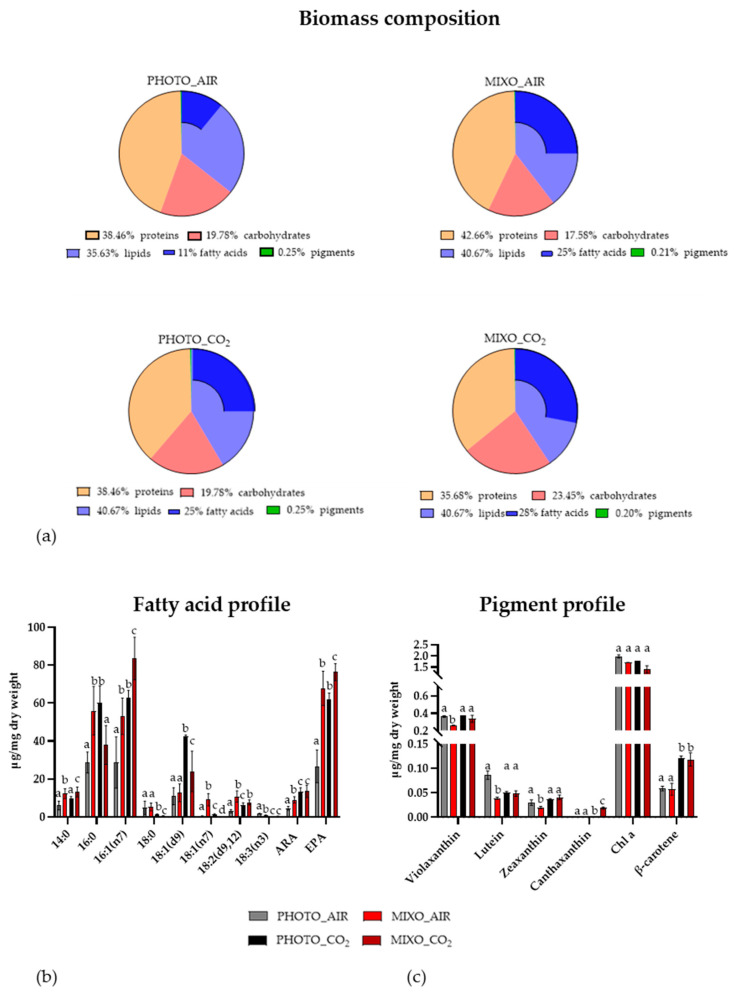
(**a**) Biomass composition, (**b**) Fatty acid, and (**c**) Pigment profile of *Nannochloropsis granulata* grown in photobioreactors with light in a medium injected with air (PHOTO_AIR); with light in a medium supplemented with 10 µM glycerol and injected with air (MIXO_AIR); with light in a medium injected with 1–2% CO_2_-enriched air (PHOTO_CO_2_); with light in a medium supplemented with 10 µM glycerol and injected with 1–2% CO_2_-enriched air (MIXO_CO_2_). Each graph shows data as mean ± standard deviation (*n* = 3–5). Different letters (a, b, c, d) denote significant differences among treatments (*t*-test, *p* < 0.05). ARA: Arachidonic acid; EPA: Eicosapentaenoic acid; Chl *a:* chlorophyll *a*.

The biomass derived from the growth of *Ng* in PBRs in the four different conditions was further analysed in terms of protein-lipid-carbohydrate composition, fatty acid, and pigment profiles. The composition of the ash-free biomass based on CHN analyses was very similar among the four tested conditions, with about 40% proteins, 40% lipids, and 20% carbohydrates (Figure 3a). However, while the fatty acid content of the PHOTO-AIR samples represented 11% of the biomass, it was more than doubled in the samples from the MIXO_AIR, PHOTO_CO_2_, and MIXO_CO_2_ conditions. Palmitic acid (C16:0), palmitoleic acid (16:1 (n7)), and EPA (20:5 (n3)) were identified as the major fatty acid classes in *Ng* (Figure 3b), as previously reported in the genus [14,21]. The amounts of most detected fatty acids were significantly increased in MIXO_AIR as compared to PHOTO_AIR, particularly the EPA that was triplicated in this condition. MIXO_CO_2_ showed the highest fatty acid content in terms of 16:1 (n7) and EPA, which were increased by 1.5- and 3-fold as compared to PHOTO_CO_2_ and PHOTO_AIR, respectively. HPLC analysis of pigment extracts from *Ng* revealed the presence of chlorophyll *a*, violaxanthin, and β-carotene as major pigments, and of lutein, zeaxanthin, and canthaxanthin as minor carotenoids (Figure 3c), thus confirming previous results for the strain [16]. In addition, the pigment profile was affected by the growth conditions. The violaxanthin, lutein, and zeaxanthin contents were significantly reduced in MIXO_AIR as compared to PHOTO_AIR. β-carotene content was doubled in PHOTO_CO_2_ and MIXO_CO_2_ as compared to PHOTO_AIR and MIXO_AIR, whereas the amount of chlorophyll a was not changed among the samples. Furthermore, canthaxanthin was significantly more concentrated in MIXO_CO_2_ (about 0.02 µg/mg of dry weight) than in PHOTO_CO_2_ (about 0.002 µg/mg of dry weight) and was not detected in the other conditions.

### 2.4. In Vitro Antiproliferative Effect on Human Cells

**Figure 4:**
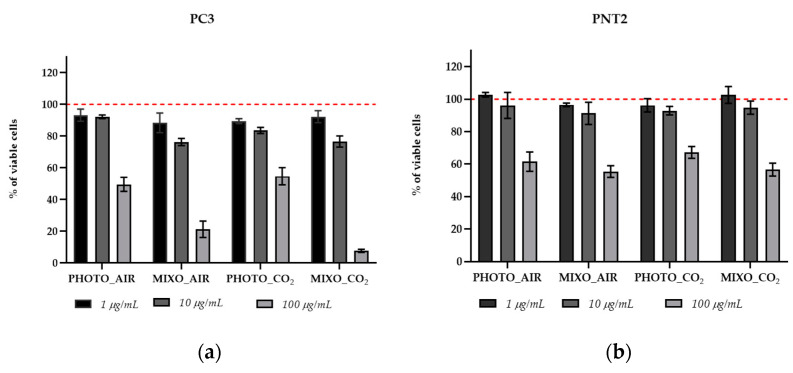
MTT cell viability assay on (**a**) PC3 prostate cancer and (**b**) PNT2 normal cell lines. Bar graphs show the percentages of viable cells after 48 h of treatment with 1, 10, and 100 μg/mL of total extracts. Cells treated with DMSO vehicle (0.5%) were used as control and correspond to 100% of cell viability (dotted red lines). Assays were performed in biological triplicates, and the graphs represent means ± standard deviations.

Total extracts of *Ng* grown in the four conditions (PHOTO_AIR, MIXO_AIR, MIXO_CO_2_, and PHOTO_CO_2_) were tested on both normal (PNT2) and cancer (PC3) human prostatic cells. These two cell lines were chosen since prostatic cancer is a very common disease in the male population, representing the second most frequent cancer type in men and the fifth leading cause of death worldwide [22]. Figure 4 shows the results of the viability assay on the two cell lines after 48 h of treatment. Cells grown under phototrophic growth (PHOTO_AIR and PHOTO_CO_2_) did not produce metabolites with a strong cytotoxic effect on PC3. Indeed, a reduction of cell viability in this cell line was induced only at the highest concentration (100 µg/mL) (49.5% for PHOTO_AIR and 54.7% for PHOTO_CO_2_). A similar effect was observed using the same extracts (PHOTO_AIR and PHOTO_CO_2_) on normal cells PNT2; 1 and 10 µg/mL showed comparable viability levels in control cells, while 100 µg/mL reduced the percentage of viable cells comparably to that observed in cancer cells (61.5% for PHOTO_AIR and 67.2% for PHOTO_CO_2_). The MIXO conditions (MIXO_AIR and MIXO_CO_2_) produced metabolites that strongly reduced the percentage of viable PC3 cells at 100 µg/mL (21.2% for MIXO_AIR and 7.6% for MIXO_CO_2_) and only a slight reduction at 10 µg/mL (76.3% for MIXO_AIR and 76.6% for MIXO_CO_2_). The same extracts (MIXO_AIR and MIXO_CO_2_) did not induce any variations at 1 and 10 µg/mL in normal PNT2 cells, and there was a reduction of cell vitality only at the highest concentration (55.4% for MIXO_AIR and 56.6% for MIXO_CO_2_).

**Figure 5:**
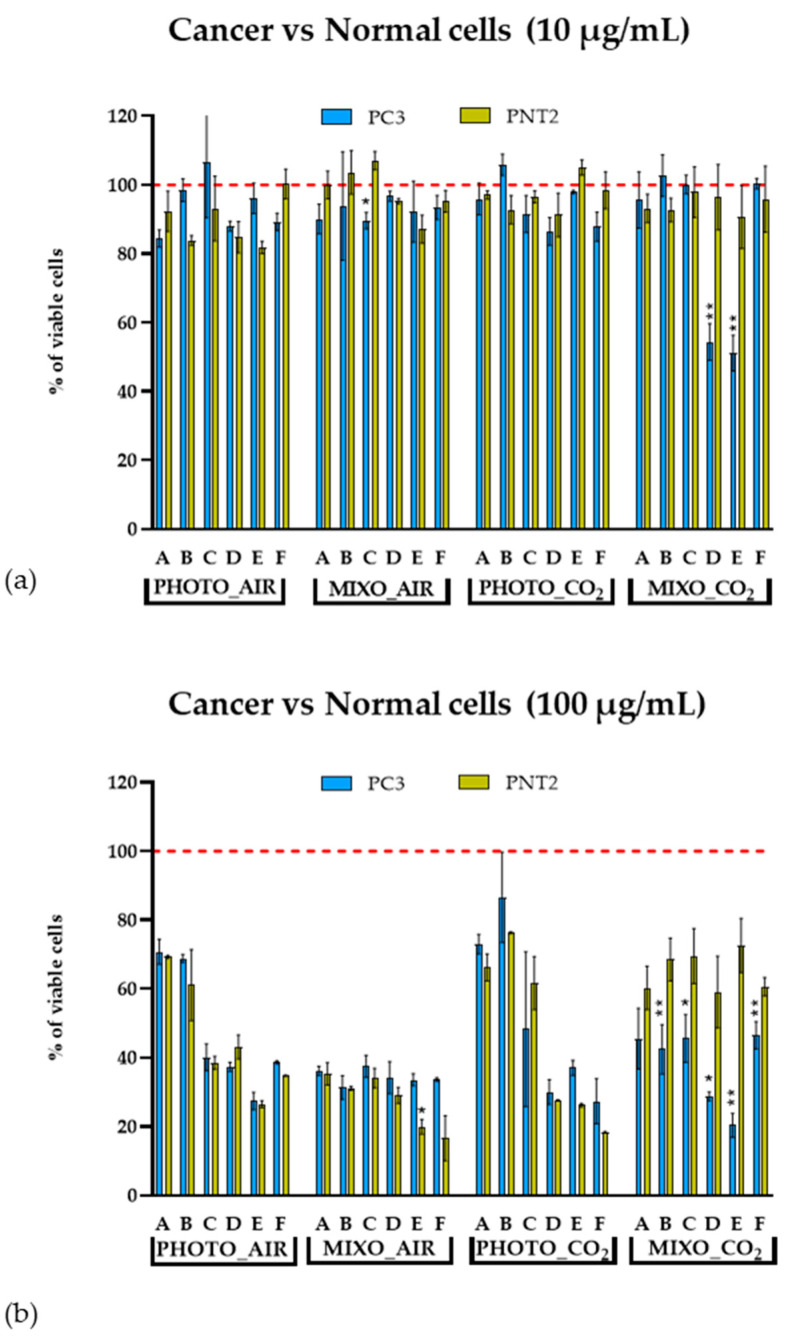
Cell viability results after treatments of PC3 (blue bar graph) and PNT2 (yellow bar graph) with all fractions (A–F). Histograms represent the percentages of viable cells after 48 h of incubation with (**a**) 10 μg/mL and (**b**) 100 μg/mL of fractions. Cells treated with DMSO vehicle (0.5%) were used as control and correspond to 100% cell viability (dotted red line). Assays were performed in biological triplicate, and graphs represent means ± standard deviations. Differences between viability of PNT2 and PC3 were considered significant for *p*-values ≤ 0.05 (** *p* ≤ 0.005 and * *p* ≤ 0.05).

Total extracts were fractionated and tested again on PC3 and PNT2. Both cell lines treated with 1 μg/mL of the fractions did not show reduction in cell viability after 48 h (see Supplementary Materials, Figure S3). The intermediate concentration (10 μg/mL) induced a significant reduction in cell viability only when PC3 cells were treated with the fractions D and E (see Section 4.6) of the MIXO_CO_2_ growth condition (Figure 5a). All fractions at 100 μg/mL induced a strong or moderate reduction in viability in both PC3 and PNT2 cells. In particular, all the fractions deriving from the PHOTO_AIR, MIXO_AIR, and PHOTO_CO_2_ extracts induced comparable levels of viability in both PC3 and PNT2, while fractions B, C, D, E, and F from MIXO_CO_2_ showed higher toxicity in PC3 as compared with PNT2 (Figure 5b).

**Figure 6:**
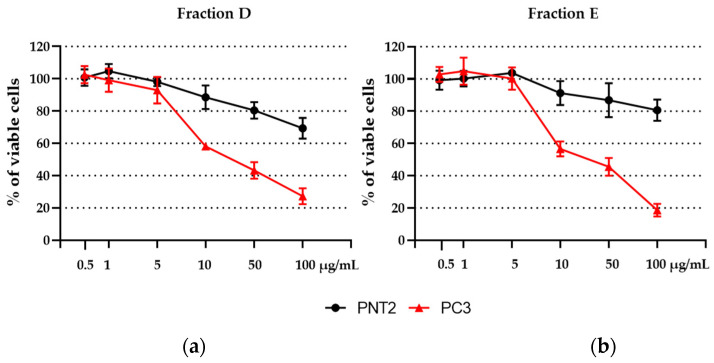
Cell viability assay on PC3 and PNT2 cells after treatment for 48 h with 0.5, 1, 5, 10, 50, and 100 μg/mL of (**a**) fraction D and (**b**) fraction E. Assays were performed in biological triplicate, and graphs represent means ± standard deviations.

Fractions D and E of MIXO_CO_2_ that showed the highest cytotoxicity in PC3 were selected since they exerted the strongest selective antiproliferative effect and were also tested with a wider range of concentrations, at both 24 h (see Supplementary Materials, Figure S4) and 48 h, for the calculation of IC50 (Figure 6). Fraction D exhibited an IC50 of 57 μg/mL while that of fraction E was 54 μg/mL; the latter was selected for cell death pathway analysis due to its lower toxicity in normal cells and its higher fractionation yield with respect to fraction D.

### 2.5. Chemical Composition of the Bioactive Fractions

Total extracts from the four different culture conditions were analysed by LC-MS in positive ion mode, thus unveiling a similar chemical profile. However, significant changes in the concentrations of the major compounds were clearly observed. Particularly, the most active extract (MIXO_CO_2_) displayed higher amounts of the metabolites eluted in the time range between 12.5 and 14.8 min (Figure S5).

**Figure 7:**
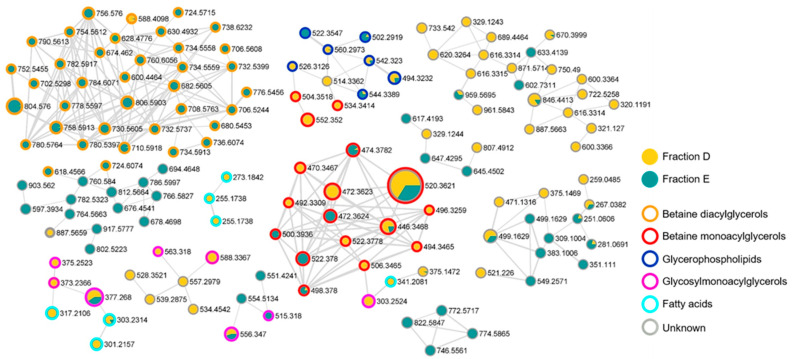
Molecular network obtained combining the LC-MS/MS analyses of fractions D and E of the MIXO_CO_2_ extract from *Nannochloropsis granulata*. Nodes are illustrated as a pie chart showing the compound source (yellow: fraction D; green: fraction E). Node size is related to metabolite amount (peak area) while edge thickness reflects cosine score similarity. The colour of the border of each node is mapped to the chemical class assigned by the molecular networking analysis of the LC-MS/MS data.

Afterwards, we performed an untargeted dereplication of the most active fractions, D and E, of the MIXO_CO_2_ crude extract for the rapid identification of compounds responsible for the antiproliferative activity. Fractions D and E were dissolved in mass grade methanol at 1 mg/mL and subjected to liquid chromatography coupled with tandem mass spectrometry (LC-MS/MS) in the data-dependent (DDA) acquisition mode. The obtained MS raw files were processed by MZmine [23] and submitted to GNPS to build a molecular network with the Feature-Based Molecular Networking (FBMN) tool [24]. In the resulting molecular network (Figure 7), each node is representative of an ion detected in one or both fractions, the size of the node is proportional to the area of the extracted ion chromatogram, while the border colour of each node is mapped to the relevant chemical class of the metabolite.

The integration of the molecular networking and MS/MS fragmentation data analysis led to the structural prediction of almost 70 metabolites from the bioactive fractions D and E, which were assigned to five chemical classes, namely betaine diacylglycerols, betaine monoacylglycerols, glycerophospholipids, glycosylmonoacylglycerols, and fatty acids (Figure 7).

Overall, betaine lipids were shown to be the major clusters detected in the bioactive fractions, with betaine diacylglycerols being almost exclusively present in fraction E and betaine monoacylglycerols being the most abundant metabolite in fraction D. Betaine lipids, structural components of cell membranes and chloroplasts, are acylglycerolipids bearing an ether-linked quaternary amine alcohol moiety at the *sn*-3 position, which may be represented by a 2′-(hydroxymethyl)-(*N*,*N*,*N*-trimethyl)-β-alanine, a carboxy-(hydroxymethyl)-choline, or a 4′-(*N*,*N*,*N*-trimethyl)-homoserine. The product ion spectra generated from the [M + H]^+^ ions of betaine lipids from fractions D and E were all characterized by the presence of the diagnostic fragment ion at *m*/*z* 236.1492 (C_10_H_22_NO_5_^+^), which is indicative of diacyl- and monoacyl-glycerol-trimethylhomoserine (DGTS and MGTS) or diacyl- and monoacyl-glyceryl-hydroxymethyl-*N*,*N*,*N*-trimethyl-β-alanine (DGTA and MGTA) [25]. Therefore, betaine lipids from the bioactive fractions were predicted to be DGTS and MGTS and/or DGTA and MGTA. Particularly, 39 DGTS/A (Table 2) and 17 MGTS/A (Table 3) were identified in fractions D and E of the MIXO_CO_2_ crude extract.

As DGTS and DGTA (as well as MGTS and MGTA) are structural isomers, and since no distinctive fragments could be observed in the tandem mass spectra of the [M + H]^+^ adducts, it was not possible to differentiate unambiguously between the structures of DGTS and DGTA (as well as MGTS and MGTA). In general, the product ion spectra of MGTS/A were dominated by the [M + H-H_2_O]^+^ daughter ion and allowed us to infer the fatty acyl substituent by the presence of the C_10_H_22_NO_5_^+^ fragment ion, which derived from the loss of the fatty acyl chain as ketene (Figure S6). On the other hand, besides the presence of the [C_10_H_22_NO_5_]^+^ fragment, the mass tandem spectra of the [M + H]^+^ ions of DGTS/A displayed fragment ions corresponding to the loss of each fatty acyl substituent at the *sn*-1 and *sn*-2 positions, both as ketene and carboxylic acid, thus giving information about their fatty acyl composition (Figure S7). The DGTS/A and MGTS/A detected in fractions D and E feature a remarkable amount of polyunsaturated fatty acids, including EPA (Table 2 and Table 3). The structural prediction of the minor molecular clusters, i.e., glycosylmonoacylglycerols (Table S1), glycerophospholipids (Table S2), and fatty acids (Table S3), is presented in the Supplementary Material.

### 2.6. Cell Death Pathway

**Figure 8:**
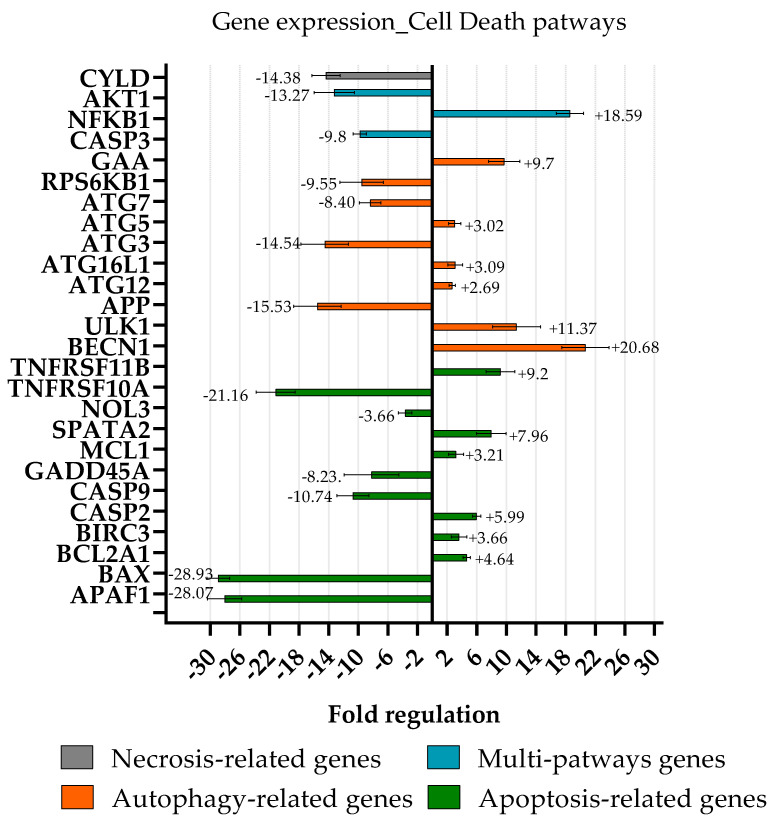
Variation of gene expression levels of death-related genes in PC3 treated with fraction E, with respect to gene expression levels of untreated PC3 cells. The p-values were calculated based on a Student’s *t*-test of the replicate 2^−ΔCT^ values for each gene in the control group and the treatment group; all fold regulations reported in this graph show *p*-values ≤ 0.05; thus, they were considered significant.

To establish the cell death signalling pathway induced by the bioactive fraction E, a comparative analysis of the gene expression levels was performed between PC3 treated with 54 μg/mL of fraction E (i.e., IC50 value) of MIXO_CO_2_ for 3 h and under control conditions (PC3 cells without any treatment, in a complete RPMI medium). Figure 8 shows the relative expression ratios of the analysed genes after treatment with respect to control. Gene expression with a threshold fold regulation value of 2 was used to select genes differentially expressed between the test and control. After a 3 h treatment of PC3, fraction E provoked a strong up-regulation of some autophagy-related genes (orange bar graph), such as ULK1 (11.4-fold change), GAA (9.7-fold change), BECN1 (20.7-fold change), ATG5 (3.0-fold change), ATG16L1 (3.1-fold change), and ATG12 (2.7-fold change). At the same time, five autophagy-related genes were down-regulated: RPS6KB1 (−9.6-fold change), CASP3 (−9.8-fold change), ATG7 (8.4-fold change), ATG3 (−14.5-fold change), and APP (−15.5-fold change). Among the anti-apoptosis genes (green bar graph), one of them was down-regulated (AKT1, −13.3-fold change) while five of them were up-regulated: TNFRSF11 (9.2-fold change), MCL1 (3.2-fold change), CASP2 (6.0-fold change), BIRC3 (3.6-fold change), and BCL2A1 (4.6-fold change). All pro-apoptosis genes found differentially expressed were down-regulated, such as TNFRSF10 (−21.2-fold change), NOL3 (−3.7-fold change), GADD45A (−8.2-fold change), CASP9 (−10.7-fold change), BAX (−28.9-fold change), and APAF1 (−28.1-fold change). Only two genes involved in the necrosis death pathway (grey bar graph) were differentially expressed; SPATA2 was up-regulated (8.0-fold change), and CYLD was down-regulated (−14.4-fold change). NFKB1, CASP3, and AKT1 are genes involved in more than one death pathway investigated (light blue bar graph); NF-κB1 was up-regulated (18.6-fold change), while CASP3 and AKT1 were found to be down-regulated (−9.8 and −13.3-fold change, respectively).

## 3. Discussion

Previous studies have investigated the mixotrophic growth of the *Nannochloropsis* genus using various external organic carbon sources [11,12,13,14], but only a few have explored the industrial potential of growing *Nannochloropsis* under this growth mode [12,14]. *Ng* has been previously proven as an industrially relevant strain due to its high biomass and its lipid productivity that is compatible with outdoor production during the summer season in Sweden [17]. In this study, we combined physiological, analytical, biological, and metabolic approaches to investigate mixotrophic growth and the biotechnological application of *Ng* grown under this trophic mode. To reduce the energetic cost required for the industrial exploitation of *Ng*, glycerol (i.e., a by-product of biofuel production) was used here as an external, low-cost carbon source. *Ng* was grown in 1L photobioreactors under four different conditions, namely phototrophy, mixotrophy, and bubbled either only with air (PHOTO_AIR and MIXO_AIR) or with CO_2_-rich air (PHOTO_CO_2_ and MIXO_CO_2_). We found that the mixotrophic growth of *Ng* in the presence of both organic and inorganic carbon (MIXO_CO_2_) was the best condition for increasing its industrial potential, as recently shown for the diatom *Phaeodactylum tricornutum* [27]. However, our results showed similar biomass productivity for *Ng* grown regardless of the presence of organic, inorganic, or both carbon sources; in contrast, a significant increase of biomass for *Phaeodactylum tricornutum* was found only in the presence of both carbon sources. Different culture conditions and different genus/species can explain these divergent results. Moreover, it was shown that a high concentration of CO_2_ inhibits the mixotrophic growth of the *Nannochloropsis* genus [13,28]. Further studies in the presence of different concentrations of CO_2_ could clarify the interaction between organic and inorganic carbon during this trophic mode in the *Ng* strain.

To investigate the industrial potential of *Ng*, the biomass composition obtained from the four different conditions was analysed. Our results showed that even if a similar macromolecular composition was maintained, the addition of either organic, inorganic, or both carbon sources (PHOTO_CO_2_, MIXO_AIR, and MIXO_CO_2_) doubled the fatty acid content as compared with the control PHOTO_AIR. This result was expected as both CO_2_ and glycerol are involved in the metabolism of fatty acids in microalgae [19,29]. The increase in fatty acid content explains the increase in calorific value, from 18 to 25 MJ/kg of dry weight in these conditions, as compared to PHOTO_AIR. A calorific value of 25 MJ/kg was also reported for *Ng* in [17] as well as for *Nannochloropsis* sp. grown in Flat-Plate Photobioreactors under N, P starvation [30], thus confirming the genus as promising feedstock for biodiesel production.

Moreover, the fatty acid profile was also affected by culture conditions, showing the increase of both palmitoleic acid (16:1 (n7)) and EPA (20:5) under mixotrophic growth, especially in MIXO_CO_2_, as compared to the phototrophic samples. The increase of EPA under mixotrophic growth using glycerol as a carbon source was already shown for *Nannochloropsis gaditana* and *Phaeodactylum tricornutum* [14,19]. EPA belongs to the class of omega-3 polyunsaturated fatty acids (PUFA) that is important for humans and animals that are not able to synthesize them and need to get them from the diet. Some microalgae, including *Nannochloropsis*, are rich in EPA, which suggests several applications in both the food and feed industries (e.g., nutraceuticals and aquaculture) [31]. The EPA content of the biomass was about 8% in the *Ng* grown in MIXO_CO_2_, and it reached state-of-the-art EPA-concentration levels as compared to others *Nannochloropsis* species grown in similar conditions [14,32]. The *Nannochloropsis* species is also a good source of valuable carotenoids such as β-carotene and canthaxanthin [16]. The increased amount of these carotenoids in the *Ng* extracts PHOTO_CO_2_ and MIXO_CO_2_ (0.1 and 0.02 µg/mg dry weight, respectively) was previously reported with the addition of glycerol and of NaHCO_3_ in other *Nannochloropsis* species [14,33]. 

*Ng* extracts displayed antitumoral activity in human prostatic cells (i.e., PC3), as previously found for different *Nannochloropsis* species tested in other human cell lines [8,9,10]. Interestingly, the PC3 cell viability under the conditions MIXO_AIR and MIXO_CO_2_ decreased from 50% to 10–20% when treated with 100 µg/mL of total extracts as compared to PHOTO_AIR and PHOTO_CO_2_. In order to further improve the antitumoral activity of *Ng*, the total extracts were fractionated using a polarity gradient elution. The most hydrophobic fractions of MIXO_CO_2_ rich in betaine lipids (i.e., D and E, 75% and 100% methanol, respectively) showed increased antiproliferative activity on cancer cells. Indeed, cell viability was already lower than 60% when the cancer cells/PC3 were exposed to 10 µg/mL of each fraction; in contrast, no cytotoxicity was exhibited (cell viability up to 90%) in the normal, prostatic PNT2 cells, as observed for the relevant total extract. In addition, fractions D and E of MIXO_CO_2_ were shown to exert highly selective antiproliferative activity towards tumor cells as compared to normal cells at 10 and 100 µg/mL. Similar results were obtained for *Nannochloropsis oculata* by [10], where the sterol-rich fractions showed the strongest antitumoral activity in HL-60 cancer cells at 24h of treatment (i.e., 50% reduction of cell viability after treatment with 25 µg/mL of the active fractions).

LC-MS/MS analysis of active fractions D and E revealed almost 70 metabolites, which were attributed to five chemical classes: betaine diacylglycerols, betaine monoacylglycerols, glycerophospholipids, glycosylmonoacylglycerols, and fatty acids. In particular, betaine lipids were the most representative classes, with MGTS/A 20:5;O_2_ (520.3621) as the most abundant metabolite of the monoacylglycerol group, and DGTS/A 20:5/20:5 (804.478) as the most abundant metabolite of the diacylglycerol group. Both betaine lipids included the polyunsaturated EPA, confirming our previous fatty acid analysis. Betaine lipids, eventually together with others, could be responsible for the antitumoral activity detected in *Ng* fractions, as observed in other organisms [34,35,36]. Finally, cell death pathways were investigated in order to understand the gene mechanism involved in the antiproliferative effect of these molecules on PC3 cells.

Almost all the genes involved in the activation or downstream process of apoptosis (APAF1 [37], BAX [38], CASP3 and 9 [39], GADD45A [40], NOL3 [41], and TNFRSF10A [42]) were downregulated, suggesting that this cell death pathway is not directly responsible for the high rate of cell death observed after the treatment of PC3. The only exception is SPATA2, a gene encoding for an adaptor protein recruited into the TNF-R1 signalling complex, which is involved in the regulation of RIPK1 [43].

Another evidence supporting the non-involvement of the apoptotic cell death is the upregulation of anti-apoptotic genes such as BCL2A1 [44], BIRC3 [45], and CASP2 [46].

The only gene involved in necrosis found to be differentially expressed was CYLD. CYLD, together with SPATA2, participates at the downstream events of TNF receptor activation and can activate apoptosis (via the CASP 8-FADD complex) or necroptosis (via the RIPK family) [47]. The downregulation of CYLD suggests that these two TNF-dependent processes are not involved in the process of cell death, also considering that only the upregulation of a single gene (SPATA2) has been observed.

Gene expression data related to autophagic factors support the activation of autophagy. GAA [48], ATG5, ATG16L1, ATG12 [49], RPS6KB1 [50], ULK1, and BECN1 [51] are involved in the activation of the autophagic process and in the formation of autophagosomes; these genes were found to be upregulated, except for RPS6KB1.

Additionally, ATG7, ATG3, and APP have been described to be involved in the autophagy cell death pathway; however, after treatment with fraction E, these three genes were downregulated. These can be explained by looking at the involvement of these three factors in the proliferation and migration of cancer cells; ATG7 [52], ATG3 [53], and APP [54] have been found to be upregulated in actively proliferating cancer cells. Thus, the downregulation of these factors together with the induction of the autophagic flux could contribute to the molecular mechanisms underlying the antiproliferative effects exerted by the lipid metabolites contained in fraction E.

To conclude, the simultaneous addition of glycerol and CO_2_ in *Ng* could be applied in outdoor systems situated along the west coast of Sweden in order to enhance the industrial potential of this strain for different applications such as biofuel, food, feed, and drug production. The use of wastewater and the optimization of the CO_2_ supply (e.g., flue gases) could further reduce the production cost and increase the biomass performance of this strain. However, the addition of organic carbon enhances the chances of competition between microalgae and complex flora [55]. Further pilot outdoor studies are needed to validate the data we obtained in the ePBRs during a simulated season on Sweden’s west coast [55].

## 4. Materials and Methods

### 4.1. Microalgae Strain and Cultivation Conditions

*Nannochloropsis granulata* (*Ng*) was initially isolated by Karlson et al. (1996) [15] from the Skagerrak, northeast Atlantic Ocean. For the experiments in this study, it was obtained from the culture collection GUMACC (Gothenburg University Marine Algal Culture Collection, https://www.gu.se/en/marina-vetenskaper/about-us/algal-bank-gumacc, accessed on 1 November 2019). This strain was selected because it was found to be the most productive local strain on the Swedish west coast in summer conditions [17]. *Ng* was not axenic, but 100 µg/L of ampicillin was added at the beginning of the cultivation in order to control the bacterial growth.

Precultures were maintained in 100 mL flasks at 16 °C, with a light intensity of about 20 μmol photons m^−2^ s^−1^ and a 12/12 h L/D (Light/Dark) cycle. The medium used was natural seawater collected from a depth of 30 m at the Tjärnö Research Station, University of Gothenburg, Sweden. The seawater was filtered using two 0.4 μm GF/F glass fibre filters, the salinity was adjusted with deionized water to 26 practical salinity units, and it was sterilized by autoclaving at 121 °C for 20 min. Finally, nutrients from the standard f/2 marine cultivation medium (NaNO_3_, NaH_2_PO_4_, microelements, vitamins [56]) were sterilized with cellulose filter paper (with pore size of 0.22 μm) and added to the autoclaved seawater.

#### 4.1.1. Screening in Multi-Cultivator

For the small-scale experiments, *Ng* was grown in a Multi-cultivator MC 1000 OD (Photon System Instruments, Check Republic) using a constant white light with an intensity of 300 µmol photons m^−2^ s^−1^, at a temperature of 20 °C, and with air bubbling (Figure S1a). These cultivation conditions were used because they correspond to the average light intensity and temperature during the summer season in Gothenburg. The Multi-Cultivator was used for small-scale growth and physiological characterisation thanks to its ability to simultaneously control growth in eight flasks containing 80 mL of liquid culture. The control was grown in phototrophic conditions without the addition of an external carbon source. For the mixotrophic conditions, 10 mM glycerol was added. All the samples were run in triplicate. The medium used was the same as that for the inoculum but using 14-fold concentrated nutrients (NaNO_3_, NaH_2_PO_4_, microelements) and vitamins in order to obtain high biomass yields. The enrichment factor was calculated from the required amounts of nutrients to obtain at least 2 g/L of biomass based on Redfield ratio for marine phytoplankton [17].

Algal growth was monitored every two days by measuring chlorophyll *a* fluorescence expressed in relative fluorescence units (RFU), using a Varioscan™ Flash Multimode Reader (Thermo Fisher Scientific, Vantaa, Finland), in 96-well microplates [17]. A total of 250 µL of samples were added into each well of the microplate (in triplicate) and incubated for 10 min in the dark. Dilutions were performed when required (i.e., chlorophyll fluorescence values > 30). Chlorophyll fluorescence was detected using a wavelength of 425 nm for excitation and 680 nm for emission. The growth profiles in the different conditions were normalized as a function of ln (RFU_t_/RFU_0_), where RFU_t_ was the chlorophyll a fluorescence at a certain time (t), and RFU_0_ was the initial chlorophyll a fluorescence. After the stationary phase was reached, the biomass yield was determined and expressed as g/L of dry weight. A total of 5 mL of final cultures was filtered through pre-weighted dried GF/F (47 mm) Whatman^®^ filters and then washed with 10 mL of 0.5 M ammonium carbonate [57]. Finally, the filters containing the culture were incubated at 100 °C for 24 h and weighted for the determination of the dry weight (biomass yield) according to the following formula:

(g of (filter + biomass)) − (g of filter)/0.005 L (volume of filtered culture)(1)

#### 4.1.2. Cultivation in Environmental Photobioreactors

Following the first screening in the Multi-Cultivator, larger-scale (i.e., 1 L of liquid culture) cultivation was carried out in photobioreactors in order to collect more biomass for further analysis (i.e., biomass composition, pigment and fatty acid profile, bioassay analysis). The inoculum of *Ng* was grown in 1 L flasks, with 500 mL of culture at room temperature, with a light intensity of 150 μmol photons m^−2^ s^−1^ and a photoperiod of 12 h light/12 h dark, bubbled with air, and stirred at 120 rpm. A total of 100 mL of cells grown for 7 days was inoculated in 900 mL of cultivation medium in environmental photobioreactors (ePBRs) (Figure S1b), corresponding to an initial OD of about 0.1 and using the spectrophotometer Thermo Scientific Evolution 60 at a wavelength of 750 nm. Dilutions were performed for samples with OD 750 nm > 1. OD was monitored throughout the growth experiment; however, only chlorophyll fluorescence (RFU) was shown in order to discriminate microalgal from bacterial growth. The cultivation medium for ePBR experiments was prepared, as described for the screening in the Multi-Cultivator. The ePBRs were programmed for “summer conditions” based on records of air temperature, light intensity, and photoperiod in Gothenburg during the summers of 2014–2016, as designed in [17]. Here, four different conditions were tested: (1) PHOTO_AIR: growth with light in a medium injected with air (i.e., 0.04% CO_2_); (2) MIXO_AIR: growth with light in a medium supplemented with 10 mM glycerol and injected with air (i.e., 0.04% CO_2_); (3) PHOTO_CO_2_: growth with light in a medium injected with 1–2% CO_2_-enriched air; (4) MIXO_CO_2_: growth with light in a medium injected with glycerol and injected with 1–2% CO_2_-enriched air. The concentration of CO_2_ in PHOTO_CO_2_ and MIXO_CO_2_ varied based on the need to maintain the pH values of cultures at 8 through the automatic injection of CO_2_. The pH of the other cultures was maintained at the same value by manual injection of 0.4 N H_2_SO_4_ when needed. The layout of the photobioreactors is modular, and the picture in Figure S1b shows one of three modules in a setup consisting of four PBRs each. The complete setup allows for 12 PBRs in total, of which nine were used for these experiments. Two treatments were used in parallel, and each treatment was allotted from three to five replicate PBRs out of the nine. All three modules were located in a temperature-controlled enclosure. Custom-built microprocessor control modules for pH, light, gas mixing, etc., were located outside the enclosure. Light was controlled separately for each PBR according to the profile in Figure 2a. pH was controlled individually for each PBR through the high-frequency pulsed addition (1 Hz control loop) of a gas mixture, i.e., filtered air or 1–2% CO_2_ in filtered air, dispersed through a capillary at the bottom of each PBR. Mixing was accomplished with magnetic stir bars set at 125 rpm. Temperature monitoring was accomplished through a temperature-controlled enclosure and by fine tuning with a water bath, which circulated water through the outer water jacket of each PBR.

Algal growth was monitored every two days by measuring chlorophyll *a* fluorescence expressed in relative fluorescence units (RFU) with the use of a fluorometer (Fluoromax 4, Jobin-Yvon, Horiba Scientific, Palaiseau, France). A total of 3 mL of samples was added in a quartz cuvette and incubated for 10 min in the dark. Dilutions were performed when required (i.e., Chlorophyll fluorescence values > 106). Chlorophyll fluorescence was detected using a wavelength of 425 nm for excitation and 680 nm for emission. The growth profiles in the different conditions were normalized, as described for the screening in Multi-Cultivator. Cultivation was stopped when the stationary phase was reached, and biomass was collected for further analyses. 

### 4.2. Photosynthetic Analysis

Photosynthetic analysis was carried out with a pulse-amplitude-modulated fluorometer DUAL-PAM 100 equipped with a DUAL-DB and a DUAL-E emitter–detector module (Walz, Effeltrich, Germany). The photosynthetic parameter variable fluorescence/maximum fluorescence (F_v_/F_m_) was determined by measuring 2 mL of a 20 min dark-adapted algae sample with the use of saturated actinic red light (300 μmol photons m^−2^ s^−1^). F_v_/F_m_ represents the maximum quantum yield of PSII and gives an indication of the physiological state of photosynthetic organisms, where F_v_ is equal to F_m_ − F_0_, and F_m_ and F_0_ are the maximum and minimum fluorescence of the dark-adapted cells, respectively.

### 4.3. Biomass Analysis

The biomass yield was determined at the end of the growth curve, when approaching the stationary phase, as described for the screening in the Multi-Cultivator. Biomass productivity was calculated by dividing the biomass yield by the number of cultivation days. The maximum specific growth rate (*μ*_max_) was calculated during the exponential phase (days 3–6) from RFU data as follows:

*µ*_max_ = (lnRFU_d2_ − lnRFU_d1_)/(d2 − d1), (2)

where RFU_d2_ and RFU_d1_ are the relative chlorophyll fluorescence on specific days of cultivation (d2 and d1).

#### 4.3.1. CHN Analysis

On the last day of cultivation, the cells were collected by centrifugation at 7000× *g* for about 20 min, stored at −80 °C, and freeze-dried for 48 h using Alpha 1–2 LD plus, Martin Christ. About 1 g of freeze-dried biomass was ground using a mortar and pestle for elemental (i.e., Carbon, Hydrogen, Nitrogen, CHN), ash content, and calorific value analyses. CHN and ash content analyses were performed using the standard methods SS-EN-ISO 16,948 with the Elemental Analyzer CHN 628, Leco and the SS-EN-ISO 18,122 with the thermogravimetric analyser TGA 701, Leco, respectively. Finally, the calorific value was determined using the standard method SS-EN-ISO 18,125 and the bomb calorimeter C5003, IKA. The ash-free biomass composition (protein, lipid, and carbohydrate content) was calculated by the equation used in [58].

The energy productivity was calculated by multiplying the biomass productivity with the calorific value.

#### 4.3.2. Fatty Acid Profile

A certain amount (5–10 µg) of freeze-dried biomass was analysed for its fatty acid content by using direct acid transmethylation and Gas Chromatography/Mass Spectrometry (GC–MS) [59]. A total of 5 µg of di-nonadecanoyl-phosphatidylcholine (C19:0) was added to the freeze-dried biomass and used as an internal standard for the quantification. An amount of 2 mL of boiling 2-propanol was then added and incubated at 100 °C for 5 min. The sample was completely evaporated under a stream of N_2_, and 1.5 mL of 2.5% H_2_SO_4_ in methanol (*v*/*v*) was added and incubated at 80 °C for 4 h and cooled down for 10 h. A total of 5 mL of 1 M cold NaCl and 1 mL of heptane were added to the samples and then mixed. The samples were centrifugated at 1000 rpm for 10 min to allow for the separation of the phases. The upper phase was collected and analysed using an Agilent 7820 GC (Agilent Technologies Co., Ltd., Shanghai, China) coupled to an Agilent 5975 MS (Agilent Technologies, Wilmington, DE, USA). The obtained fatty acid methyl esters (FAMEs) were separated on a 30 m × 0.25 mm DB-23 capillary column (Agilent), using helium as a carrier gas at a constant flow of 0.6 mL min^−1^ and a temperature of 210 °C. The FAMEs were identified by their comparison with commercial standards from Sigma-Aldrich, Darmstadt, Germany (Me 100, Me81, and individual FAME, Larodan, and Marine PUFA no.3) and quantified by comparison with the internal standard (C19:0). The concentration of FAMEs was then normalized for the freeze-dried biomass and expressed as µg/mg of dried weight.

#### 4.3.3. Pigment Profile

A certain amount of freeze-dried (2–6 µg) biomass was resuspended in 5 mL of 90% (*v*/*v*) acetone contained in falcon tubes covered with aluminium foil to prevent the entry of light. The samples were ground in a glass homogenizer and refrigerated at 4 °C for 4 h. After the incubation period, the samples were centrifuged at 3000 rpm for 5 min. Up to 5 mL of the clear supernatant with 90% acetone was taken and used for pigment quantification. The samples were then filtered using a filter with a pore size of 0.2 µm prior to run. The pigment composition of the samples was obtained using HPLC PDA analysis. The samples were analysed in a Shimadzu UFLC system (Shimadzu corporation, Kyoto, Japan) loaded with an Alltima C18 (RP18, ODS, Octadecyl) 150 × 4.6 mm column, using 100 µL injection volume. The carotenoids and chlorophyll a were eluted through a low-pressure gradient system comprised of Solvent A with methanol and 0.5 M ammonium acetate buffer (85:15), solvent B with acetonitrile and milliQ water (90:10), and solvent C with 100% ethyl acetate. The program consisted of solvent 100% B:0% C: (8 min), 90% B:10% C: (8.6 min), 65% B:35% C (13.1 min), 31% B:69% C (21 min), and 100% B:0% C (27 min). Retention time and spectra obtained from standards (DHI, Hørsholm, Denmark) and run under the same conditions were used to identify the carotenoids in the samples. The pigment concentration was obtained using quantification based on the area of each standard. The pigment concentration was then normalized for freeze-dried biomass and expressed as µg/mg of dried weight.

### 4.4. Nutrient Analysis

Every 2 days, about 2 mL of growing cultures were filtrated using a nylon filter with a pore size of 0.22 μm. The samples were diluted 20 times with MilliQ water and stored at 4 °C until the nitrate (N) and phosphate (P) analysis. The diluted media were then analysed using the ion chromatographic system 882 Compact IC plus, coupled with the 858 Professional Sample Processor Metrohm AG, Herisau, Switzerland, and the anion exchange chromatographic column Metrosep Asup 5–250/4.0, Metrohm AG, Herisau, Switzerlan, using a conductivity detector (Metrohm AG, Herisau, Switzerland, part number 2.850.9010). In this analysis, an injection volume of 100 µL and an eluent of 3.2 mM Na_2_CO_3_ and 1 mM NaHCO_3_ pumped at 0.7 mL/min were used.

The glycerol analysis was conducted using the same ion chromatographic system described above after replacing the column and detector with a Metrosep Carb 2-150/4.0 column and an IC amperometry detector (Metrohm AG, Herisau, Switzerland, part number 2.850.9110). The detector was used with palladium/gold electrodes in the pulsed amperometry mode. The injection volume was 20 µL, flowrate was at 0.5 mL min^−1^, and 100 mM NaOH/10 mM sodium acetate was used as eluent.

N, P and glycerol (Gly) removal rates were calculated with the difference between the initial and final concentrations of these nutrients in the media, divided by the days of the experiment (d), and expressed as mg/mL/d.

### 4.5. Statistical Analysis

The biomass analysis and nutrient consumption for mixotrophic and phototrophic growth were compared by *t*-test analysis using GraphPad 9.3.1 Software 2365 Northside Dr. Suite 560, San Diego, CA 92108, USA. *p*-values were used to quantify the variability between the four different growth conditions. Data were considered significant for *p*-values < 0.05.

### 4.6. Extraction, Fractionation, and Liquid Chromatography—Mass Spectrometry

Freeze-dried biomasses obtained from the four growth conditions were re-suspended in 100% methanol and homogenised by using a glass pestle. Methanol (MeOH) was chosen for the extraction as it is a non-selective solvent and allows for the extraction of the largest possible number of metabolites with a wide polarity range [60]. The samples were kept in agitation and dark conditions for 60 min at room temperature, allowing for the complete extraction of intracellular metabolites. After this step, the samples were centrifuged at 6000× *g* for 10 min at 4 °C in order to discard cellular structures and collect only the supernatants. The supernatants were dried in a rotary evaporator to obtain the dried total extracts to be used for chemical analysis, bioactivity assays, and fractionation. Dried total extracts were re-suspended in methanol at 100 mg/mL and loaded onto the SPE polypropylene column CHROMABOND^®^ C18 ec (column volume 6 mL, filling quantity 1000 mg) assembled on an SPE Vacuum system. The total extracts were separated into six fractions using methanol for a polarity gradient elution: fraction A, 100% H_2_O; fraction B, 25% MeOH and 75% H_2_O; fraction C, 50% MeOH and 50% H_2_0; fraction D, 75% MeOH and 25% H_2_O; fraction E, 100% MeOH; fraction F, 100% MeOH containing 1% of trifluoroacetic acid. All eluted fractions were dried in a rotary evaporator and stored at −20 °C for further chemical and biological analyses.

In order to outline a metabolomic overview, the crude extracts from the four different culture conditions, i.e., MIXO_AIR, MIXO_CO_2_, PHOTO_AIR, and PHOTO_CO_2_, were dissolved in mass grade MeOH at a concentration of 1 mg/mL and analysed by LC/MS, with a mass/charge range of 150–1000 *m*/*z* on a QTRAP 4500 (SCIEX, Framingham, MA, USA) connected to a Nexera X2 UHPLC (Shimadzu, Kyoto, Japan), which was equipped with a 1.7 m Acquity UPLC BEH C18 column (2.1 × 50 mm). For these experiments, the ESI source was set in positive mode, with the voltage set at 4.5 kV and the capillary temperature at 285 °C. Buffer A (H_2_O + 0.1% Formic acid (FA)) and Buffer B (ACN 0.1% + FA) were used for the chromatographic separation, which was executed by adopting the following gradient: from 100% A to 100% B in 20 min, followed by 5 min of 100% B.

The LC-HRMS/MS data-dependent analyses (DDA) of the SPE fractions D and E were carried out on a Thermo LTQ Orbitrap XL (Thermo Fisher Scientific, Waltham, MA, USA) with ESI source coupled to a Thermo U3000 HPLC system equipped with a 5 m Kinetex C18 column (2.1 × 50 mm). The DDA LC-MS/MS experiments were conducted by dissolving the SPE fractions at 1 mg/mL in mass grade MeOH. The gradient program was set as follows: 10% MeOH 1 min, 10−100% MeOH over 30 min, 100% MeOH 10 min. Mass spectra were acquired in the positive ion detection mode. MS parameters were as follows: a spray voltage of 4.8 kV, a capillary temperature of 285 °C, a sheath gas rate of 32 units N_2_ (ca. 150 mL/min), and an auxiliary gas rate of 15 units N_2_ (ca. 50 mL/min). Data were collected in the DDA mode, in which the five most intense ions of a full-scan mass spectrum were subjected to HRMS^2^ fragmentation. The m/z range for DDA was set between 150 and 2000 amu. HRMS^2^ scans were obtained with CID fragmentation, an isolation width of 2.0, normalized collision energy of 35, activation Q of 0.250, and an activation time of 30 ms.

### 4.7. Antiproliferative Screening and Dose-Response Curve

Antiproliferative experiments were performed on two different cell lines: PC3—human prostate adenocarcinoma (purchased from the American Type Culture Collection, ATCC, product code: CRL-1435™) and PNT2—human normal prostate epithelium immortalized with SV40 (purchased from the Sigma Aldrich, Burlington, MA, USA; product code: 95012613). The selection of a cancer cell line and its normal counterpart is essential as it reveals a selective antiproliferative activity towards cancer cells that has been induced by treatments. PC3 and PNT2 cells were grown in an RPMI (Roswell Park Memorial Institute) medium 1640 completed with 10% FBS (Fetal Bovine serum). Penicillin (100 units/mL) and streptomycin (100 µg/mL) were added to the cell medium. Cells were grown in a 5% CO_2_ atmosphere at 37 °C and allowed to reach a maximum confluence of 80% in cell culture flasks with vented filter caps. Before the treatments, the cells were harvested with trypsin (1X), counted, seeded in 96-well plates (2 × 10^4^ cells × well^−1^, with a final volume of 100 µL for each well), and incubated in 5% CO_2_ atmosphere at 37 °C overnight. Total extracts (four dried methanolic extracts of microalgal biomasses obtained from the MIXO_AIR, MIXO_CO_2_, PHOTO_AIR, and PHOTO_CO_2_ growth conditions) and fractions (six dried fractions obtained after SPE fractionation of each total extract) were dissolved in dimethyl sulfoxide (DMSO) and used for all cell treatments. The final concentration of the DMSO used was 0.5% (*v*/*v*) for each treatment. Cells were treated in biological triplicate (three technical replicates were set up for each biological replicate) with all samples (total extracts and fractions) at 1, 10, and 100 µg/mL, for 48 h in a complete cell medium. Two fractions (D and E, Mixo_CO_2_) were selected for a further viability assay since they were able to induce the strongest and most selective antiproliferative effect on prostatic cancer cells; thus, they were used to set up a dose–response curve on both cell lines. In this case, the concentrations used were 0.5, 1, 5, 10, 50, and 100 µg/mL, for 24 and 48 h. Control cells were incubated in a complete cell medium with 0.5% of DMSO for all experiments.

#### Cell Viability

The antiproliferative effect of the samples on cell viability was evaluated using the 3-(4,5-Dimethylthiazol-2-yl)-2,5-diphenyl tetrazolium bromide (MTT) assay. Briefly, at the end of the incubation of the PC3 and PNT2 cells, with the total extracts and all fractions for antiproliferative screening and fractions D and E for the dose–response curve (see Section 4.7), cell culture media (complete RPMI media containing extracts and fractions at different concentrations) were discarded from 96-well plates using a vacuum aspirator system. In each well, fresh media containing 5 µg/mL of the MTT solution were added. Plates were Incubated in a 5% CO_2_ atmosphere for 3 h at 37 °C. After incubation, the MTT solution was removed using a vacuum aspirator system, and formazan salts produced by viable cells were dissolved in an isopropanol solution (100 µL) and incubated at room temperature for 30 min on an orbital shaker. The absorbance of each well was read at 570 nm using an Infinite M1000Pro (TECAN, Männedorf, Switzerland) plate reader. The antiproliferative effect of the extracts and fractions at different concentrations was reported as percent of cell viability, calculated as the ratio between the mean absorbance of each treatment and the mean absorbance of the control (cells treated with only 0.5% of DMSO).

### 4.8. LC-HRMS2 Data Processing and Molecular Networking

LC-HRMS^2^ data from the bioactive fractions D and E were processed together to generate a molecular network, using a previously reported method [61,62]. MS raw files were imported into MZmine 2.53 [23]. Mass detection from raw files was performed for mass levels 1 and 2 by setting the noise level at 1000 and 100, respectively. Chromatograms were built by using the ADAP chromatogram algorithm, setting a minimum height of 1000 and an *m*/*z* tolerance of 0.05 (or 20 ppm). The baseline cut-off algorithm was employed for chromatogram deconvolution with the following parameters: minimum height peak = 1000, peak duration range = 0.0–10.0 min, baseline level = 100, *m*/*z* range for MS^2^ scan = 0.05, retention time range = 0.5 min. Chromatogram peaks were aligned by the Join aligner algorithm with the following settings: *m*/*z* tolerance at 0.05 or 20 ppm, absolute RT tolerance at 0.5 min. Peaks without associated MS tandem spectra were removed from the peak list. Processed mass data were exported to mgf file and submitted to the Feature Based Molecular Networking (FBMN) tool to generate the molecular network depicted in Figure 7. FBMN parameters were set as follows: precursor ion mass tolerance = 0.02, fragment ion mass tolerances = 0.02 Da, cosine score ≥ 0.7, minimum matched fragment ions = 3. The molecular network was visualized in Cytoscape version 3.7.2 (Cytoscape Consortium, San Diego, CA, USA) [63]. Chromatographic data were exported as a csv file from processed LC/MS data by MZmine and then mapped to the relevant nodes in the generated network (Available online: https://gnps.ucsd.edu/ProteoSAFe/status.jsp?task=d0d43c1f93cc4a5db75020e59299334c (accessed on 4 April 2022).

### 4.9. RNA Extraction and RT^2^ Profiler PCR Array

Prior to the RNA extraction, PC3 cells were seeded in 6-well culture plates (2 × 10^5^ cells × well^−1^, with a final volume of 3 mL for each well) using a complete RPMI medium and incubated in 5% CO_2_ atmosphere at 37 °C overnight. PC3 cells were treated with 52 μg/mL (corresponding to IC50 concentration) of active fraction E; control condition was also set up using PC3 cells in a complete RPMI medium. After 3 h of treatment, media were discarded from control and treated PC3 cells using a vacuum aspirator system, and then PC3 cells were washed directly into the wells by adding PBS and rocking gently. PC3 cells (control and treated) were lysed directly into the wells by adding 0.5 mL × well of Trisure Reagent (Bioline). RNA was isolated according to the manufacturer’s protocol. RNA concentration and purity were assessed using the nanophotomer Nanodrop (Euroclone). A total of 400 ng of RNA was subjected to reverse transcription reaction using the RT^2^ first strand kit (Qiagen, cat. 330401, Hilden, Germany) according to the manufacturer’s instructions. Real-time quantitative reverse transcription-PCR (qRT-PCR) was performed in biological triplicate using the RT^2^ Profiler PCR Array kit (Qiagen, cat.330231) in order to analyse the expression of 84 cell death genes in PC3 after exposure to the active fraction E. Plates were run on a ViiA7 (Applied Biosystems 384 well blocks, Waltham, MA, USA), Standard Fast PCR Cycling protocol with 10 μL reaction volumes. The cycling conditions used were 1 cycle initiation at 95.0 °C for 10 min, followed by amplification for 40 cycles at 95.0 °C for 15 s and 60.0 °C for 1 min. Amplification data were collected with the ViiA 7 RUO Software (Applied Biosystems). Ct values were analysed with the Qiagen data analysis online software (Available online: https://geneglobe.qiagen.com/it/analyze (accessed on 22 December 2021).

## Figures and Tables

**Table 1:** Maximum specific growth rate (*μmax*), biomass yield, biomass productivity, calorific value, energy productivity, nutrient removal rate (N, P, and Gly), and ash content were calculated for *Nannochloropsis granulata* grown in photobioreactors, with light in a medium injected with air (PHOTO_AIR); with light in a medium supplemented with 10 mM glycerol and injected with air (MIXO_AIR); with light in a medium injected with 1–2% CO_2_-enriched air (PHOTO_CO_2_), and with light in a medium supplemented with 10 µM glycerol and injected with 1–2% CO_2_-enriched air (MIXO_CO_2_). Each parameter is expressed as mean ± standard deviation (*n* = 3–5). Different letters (^a^, ^b^, ^c^) denote significant differences among treatments (*t*-test, *p* < 0.05). P: phosphate, N: nitrate, Gly: glycerol, DW: dry weight, n.a.: not applicable.

Parameters	PHOTO_AIR	MIXO_AIR	PHOTO_CO_2_	MIXO_CO_2_
Maximum specific growth rate (μ_max_, d^−1^)	0.26 ± 0.02 ^a^	0.42 ± 0.05 ^b^	0.46 ± 0.01 ^b^	0.41 ± 0.02 ^b^
Biomass yield (g DW/L)	0.90 ± 0.14 ^a^	3.23 ± 0.25 ^b^	3.36 ± 0.38 ^b^	3.38 ± 0.82 ^b^
Biomass productivity (g DW/L/d)	0.05 ± 0.01 ^a^	0.18 ± 0.01 ^b^	0.19 ± 0.02 ^b^	0.19 ± 0.05 ^b^
Calorific value (MJ/kg DW)	18.20 ± 0.80 ^a^	24.90 ± 0.99 ^b^	24.60 ± 1.20 ^b^	24.50 ± 0.71 ^b^
Energy productivity (kJ/L/d)	0.93 ± 0.15 ^a^	4.47 ± 0.39 ^b^	4.14 ± 0.58 ^b^	4.60 ± 1.13 ^b^
P removal rate (mg/L/d)	1.84 ± 0.26 ^a^	6.72 ± 1.03 ^b^	3.07 ± 0.30 ^c^	7.58 ± 1.70 ^b^
N removal rate (mg/L/d)	14.90 ± 0.30 ^a^	63.26 ± 2.76 ^b^	63.61 ± 4.30 ^b^	60.30 ± 5.40 ^b^
Gly removal rate (mg/L/d)	n.a.	61.67 ± 9.93 ^a^	n.a.	68.77 ± 8.40 ^a^
Ash content (% DW)	25.00 ± 1.20 ^a^	8.02 ± 0.20 ^b^	7.95 ± 0.49 ^b^	6.70 ± 1.40 ^b^

**Table 2:** DGTS/A betaine lipids identified by molecular networking analysis of LC-MS/MS data from the bioactive fractions D and E of the MIXO_CO_2_ crude extract (mass error < 3 ppm).

*m*/*z*	[M + H]^+^	*R*_t_	Fatty Acyl Chains ^1^
674.4620	C_39_H_64_O_8_N	31.3	9:1;O/20:5
600.4464	C_33_H_62_O_8_N	31.3	9:1;O/14:0
588.4098	C_31_H_58_O_9_N	31.3	5:1;O2/16:0
628.4776	C_35_H_66_O_8_N	32.6	9:1;O/16:0
630.4932	C_35_H_68_O_8_N	32.7	9:0;O/16:0
734.5558	C_43_H_76_O_8_N	34.4	16:0/17:4;O
780.5397	C_47_H_74_O_8_N	34.5	17:4;O/20:5
706.5244	C_41_H_72_O_8_N	34.6	16:2/17:4;O
732.5399	C_43_H_74_O_8_N	34.7	16:1/17:4;O
724.5715	C_42_H_78_O_8_N	34.8	16:0/16:2;O
776.5456	C_48_H_74_O_7_N	35.0	18:5/20:5
702.5298	C_42_H_72_O_7_N	35.1	12:0/20:5
752.5455	C_43_H_78_O_9_N	35.2	16:4/20:5
734.5559	C_43_H_76_O_8_N	35.2	16:0/17:4;O
778.5597	C_48_H_76_O_7_N	35.4	18:4/20:5
790.5613	C_49_H_76_O_7_N	35.4	19:5/20:5
754.5612	C_46_H_76_O_7_N	35.5	16:2/20:5
804.5760	C_50_H_78_O_7_N	35.6	20:5/20:5
680.5453	C_40_H_74_O_7_N	35.6	14:0/16:2
780.5764	C_48_H_78_O_7_N	35.7	18:3/20:5
730.5605	C_44_H_76_O_7_N	35.7	14:0/20:5
706.5608	C_42_H_76_O_7_N	35.8	16:2/16:1
756.5760	C_46_H_78_O_7_N	35.8	16:1/20:5
806.5903	C_50_H_80_O_7_N	35.9	20:5/20:4
682.5605	C_40_H_76_O_7_N	36.0	14:0/16:1
782.5917	C_48_H_80_O_7_N	36.0	18:2/20:5
732.5737	C_44_H_78_O_7_N	36.1	14:0/20:4
708.5763	C_42_H_78_O_7_N	36.1	14:0/18:2
708.5763	C_42_H_78_O_7_N	36.1	16:1/16:1
734.5913	C_44_H_80_O_7_N	36.3	16:1/18:2
734.5913	C_44_H_80_O_7_N	36.3	16:0/18:3
734.5913	C_44_H_80_O_7_N	36.3	14:0/20:3
758.5913	C_46_H_80_O_7_N	36.4	16:0/20:5
784.6071	C_48_H_82_O_7_N	36.5	18:1/20:5
710.5918	C_42_H_80_O_7_N	36.7	16:1/16:0
760.6056	C_46_H_82_O_7_N	36.8	16:0/20:4
736.6074	C_44_H_82_O_7_N	37.0	16:1/18:1
736.6074	C_44_H_82_O_7_N	37.0	16:0/18:2
724.6074	C_43_H_82_O_7_N	37.4	16:0/17:1

^1^ Fatty acids have been reported using the LIPID MAPS shorthand notation [26]. Fatty acyl chains are indicated as C:N;O, where C is the number of carbon atoms, N is the number of double bond equivalents, and O is the number of additional oxygen atoms linked to the hydrocarbon chain.

**Table 3:** MGTS/A betaine lipids identified by molecular networking analysis of LC-MS/MS data from the bioactive fractions D and E of the MIXO_CO_2_ crude extract (mass error < 3 ppm).

*m*/*z*	[M + H]^+^	*R*_t_	Fatty Acyl Chain ^1^
534.3414	C_30_H_48_O_7_N	25.5	20:6;O
504.3518	C_26_H_50_O_8_N	25.7	16:1;O2 ^2^
496.3259	C_27_H_46_O_7_N	26.1	17:5;O
552.3520	C_30_H_50_O_8_N	26.5	20:5;O2
492.3309	C_28_H_46_O_6_N	27.8	18:5
506.3465	C_29_H_48_O_6_N	28.8	19:5
494.3465	C_28_H_48_O_6_N	28.8	18:4
470.3467	C_26_H_48_O_6_N	28.9	16:2
446.3468	C_24_H_48_O_6_N	29.4	14:0
472.3624	C_26_H_50_O_6_N	29.4	16:1
520.3621	C_30_H_50_O_6_N	29.7	20:5
472.3623	C_26_H_50_O_6_N	30.0	16:1
522.3780	C_30_H_52_O_6_N	30.1	20:4
498.3780	C_28_H_52_O_6_N	30.6	18:2
522.3778	C_30_H_52_O_6_N	30.7	20:4
474.3782	C_26_H_52_O_6_N	31.3	16:0
500.3936	C_28_H_54_O_6_N	31.7	18:1

^1^ Fatty acids have been reported using the LIPID MAPS shorthand notation [26]. Fatty acyl chains are indicated as C:N;O, where C is the number of carbon atoms, N is the number of double bond equivalents, and O is the number of additional oxygen atoms linked to the hydrocarbon chain. ^2^ MGTS/A with a putative hydroperoxyhexadecenoic acid as a fatty acyl substituent, as revealed by a neutral loss of 34.0055 Da from the [M + H]^+^ ion, arising from fragmentation of the hydroperoxy group.

## Supplementary Materials

The following supporting information can be downloaded at: https://www.mdpi.com/article/10.3390/md20070424/s1, Figure S1: Cultivation systems used in the experiments. Figure S2: Growth profile of *Nannochloropsis granulata* under mixotrophy and phototrophy; Figure S3: Cell viability results after treatments of PC3 and PNT2 with 1 μg/mL of fractions (A–F); Figure S4: Cell viability assay on PC3 and PNT2 cells after treatment for 24 h with fraction D and E; Figure S5: Base peak chromatograms of crude extracts from *Nannochloropsis granulata*. Figure S6: HR ESI-MS^2^ spectra of the [M + H]^+^ ion of MGTS/A 20:5; Figure S7: HR ESI-MS2 spectra of the [M + H]^+^ ion of DGTS/A 14:0/20:5; Table S1: Glycosylmonoacylglycerols from the bioactive fractions D and E from *Nannochloropsis granulata*; Table S2: Glycerophospholipids from the bioactive fractions D and E from *Nannochloropsis granulata*; Table S3: Fatty acids from the bioactive fractions D and E from *Nannochloropsis granulata*. Table S4: List of all cell death genes analysed and involved in apoptosis, necrosis, and autophagy. Reference [64] are cited in the supplementary materials.
