# Supplementary material for: Mixotrophy in a Local Strain of Nannochloropsis granulata for Renewable High-Value Biomass Production on the West Coast of Sweden
Source: Mar Drugs. 2022 Jun 28;20(7):424. doi: 10.3390/md20070424 (PMC9316773; doi:10.3390/md20070424)
Supplement: Supplementary file 1 [file marinedrugs-20-00424-s001.zip › marinedrugs-1758179-supplementary.pdf]

marine drugs

Supplementary Material

Mixotrophy in a local *Nannochloropsis* strain for renewable high-value biomass production on the west coast of Sweden

Table of Contents

Figures

Figure S1. Cultivation systems used in the experiments. p.2

Figure S2. Growth profile of *Nannochloropis granulata* under mixotrophy and phototrophy..... p.3

Figure S3. Cell viability results after treatments on PC3 and PNT2 with 1 μg/mL of fractions. p.3

Figure S4. Cell viability assay on PC3 and PNT2 cells after treatment for 24 hours p.4

Figure S5. Base peak chromatograms of crude extracts from *Nannochloropsis granulata*............ p.4

Figure S6. HR ESI-MS^2 spectra of the $[M+H]^+$ ion of MGTS/A 20:5... p.5

Figure S7. HR ESI-MS^2 spectra of the $[M+H]^+$ ion of DGTS/A 14:0/20:5 .... p.6

Tables

Table S1. Glycosylmonoacylglycerols from the bioactive fractions D and E.............................. p.7

Table S2. Glycerophospholipids from the bioactive fractions D and E.. p.7

Table S3. Fatty acids from the bioactive fractions D and E p.8

Table S4. List of all cell death genes analysed p.8-11

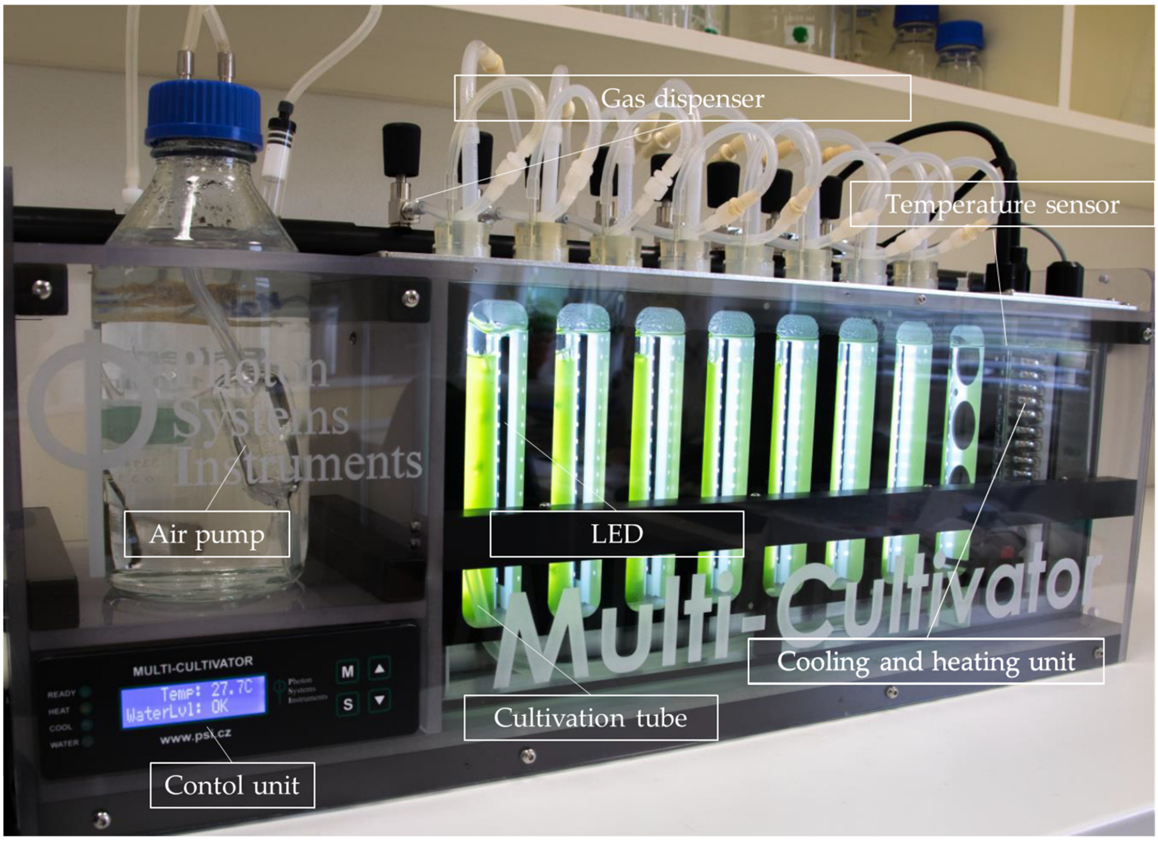

(a)

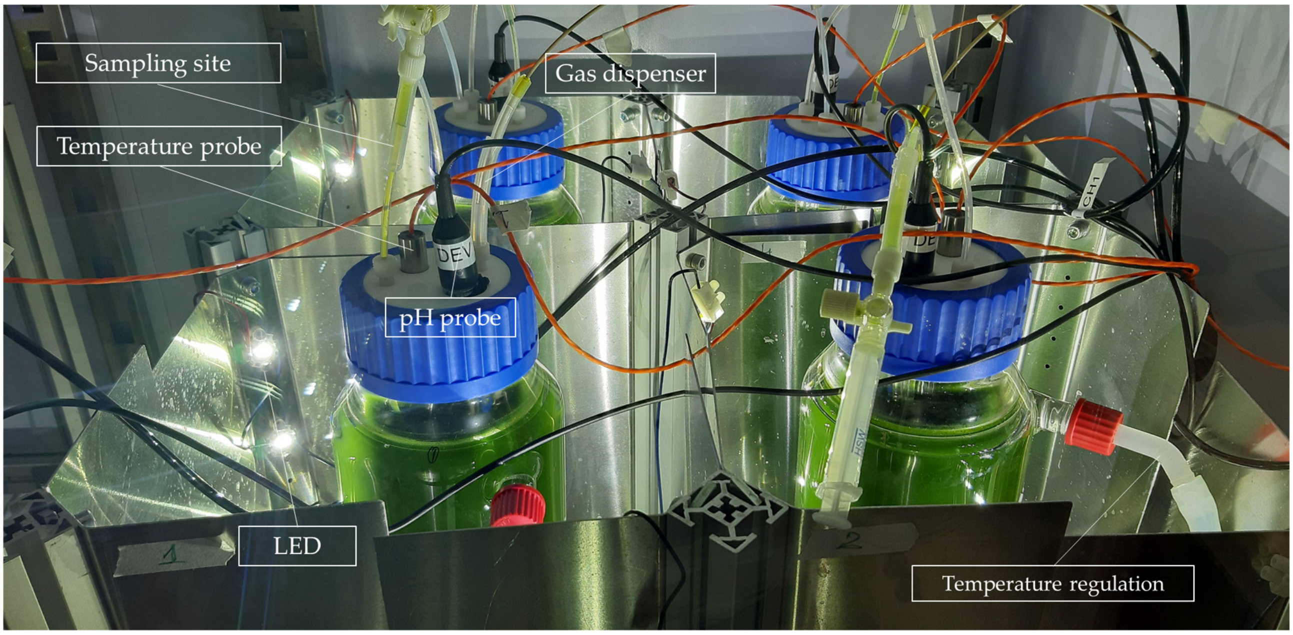

(b)

Figure S1. Cultivation systems used in the experiments; (a) Multi-cultivator MC 1000 OD (Photon System Instruments, Check Republic) where 8 cultivation flasks containing 80 mL liquid culture of Ng were run in parallel. A digital control unit allowed to control temperature, light and aeration in all the samples; (b) environmental photobioreactors (ePBRs) where 9 samples (of which 4 are showed in the picture) containing 1 L liquid culture of Ng were run in parallel. Light, pH and gas mixing were controlled separately for each ePBR through custom-built microprocessor control modules located outside the enclosure. Temperature control is accomplished by a temperature-controlled enclosure and fine control with a circulating water bath, which circulates water through the outer water jacket of each ePBR.

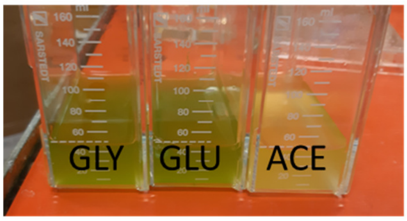

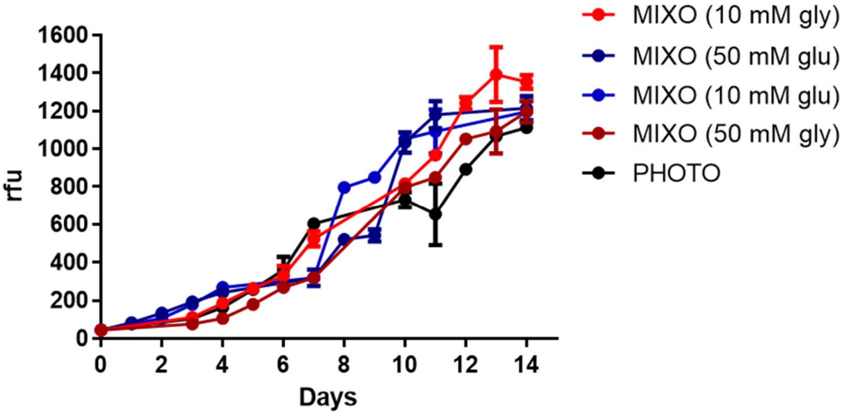

(**a**) (**b**)

Figure S2. (a) Preculture grown in flasks in presence of glycerol (GLY), glucose (GLU), and acetate (ACE); (b) growth profile of *Nannochloropsis granulata* grown in multicultivator in mixotrophy using two concentration (10, 50 mM) of glycerol and glucose and in phototrophy.

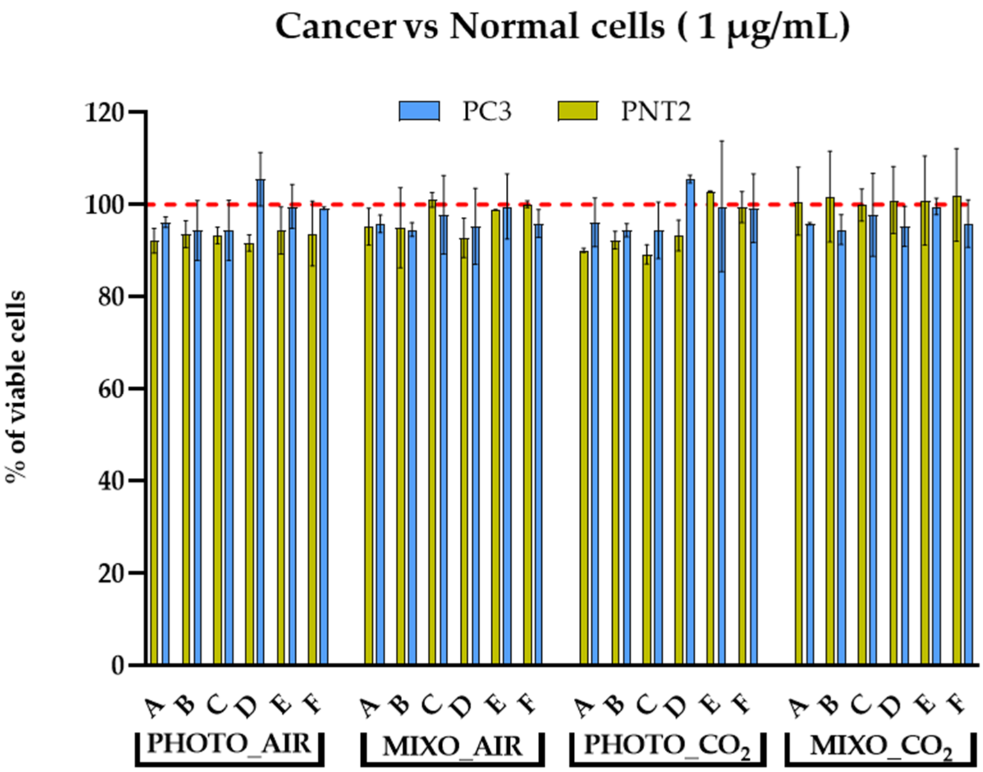

Figure S3. Cell viability results after treatments on PC3 and PNT2 with 1 μg/mL of fractions (A-F)

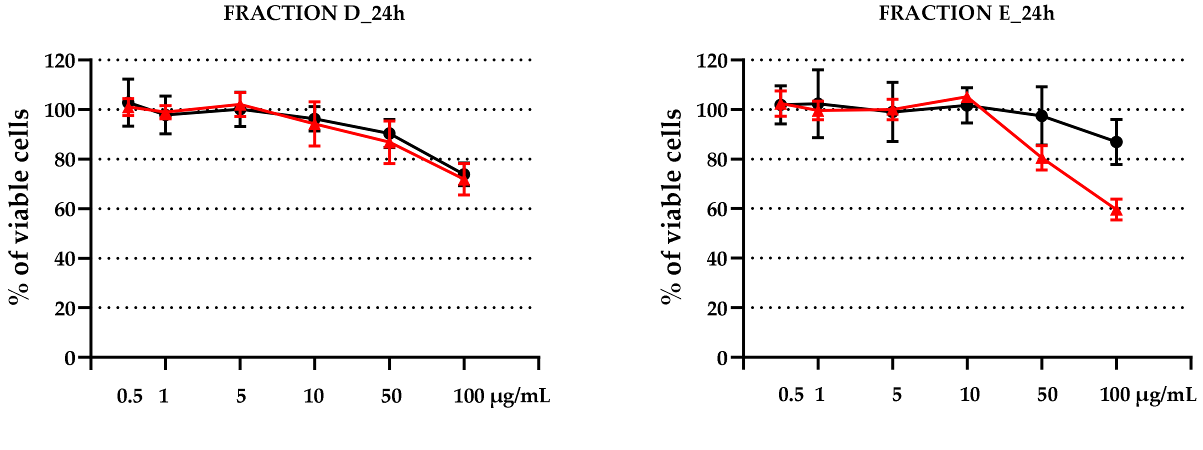

(**a**) (**b**)

Figure S4. Cell viability assay on PC3 and PNT2 cells after treatment for 24 hours with 0.5, 1, 5, 10, 50 and 100 µg/mL of (a) fraction D and (b) fraction E. Assays were performed in biological triplicate and graphs present means ± standard deviations.

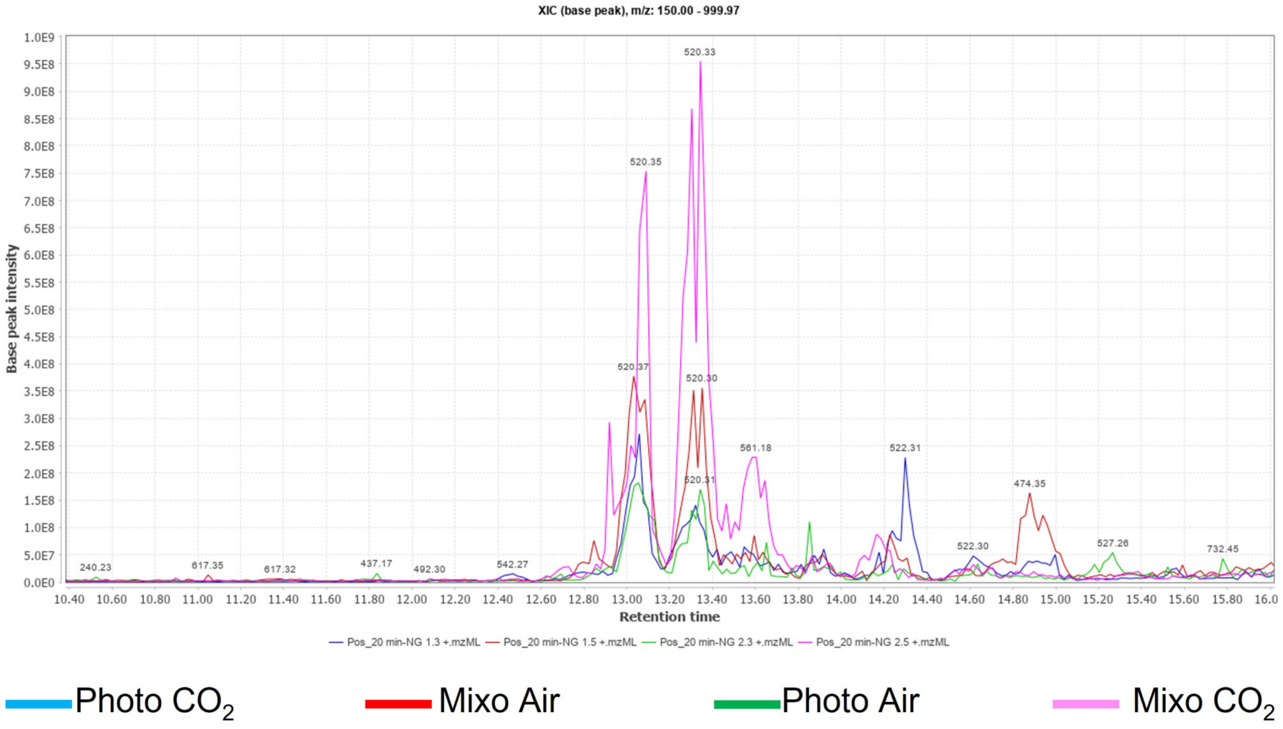

Figure S5. Base peak chromatograms of crude extracts from *Nannochloropsis granulata* cultivated under four different conditions, i.e. PHOTO_ CO_2, MIXO_CO_2, MIXO_AIR, and PHOTO_AIR.

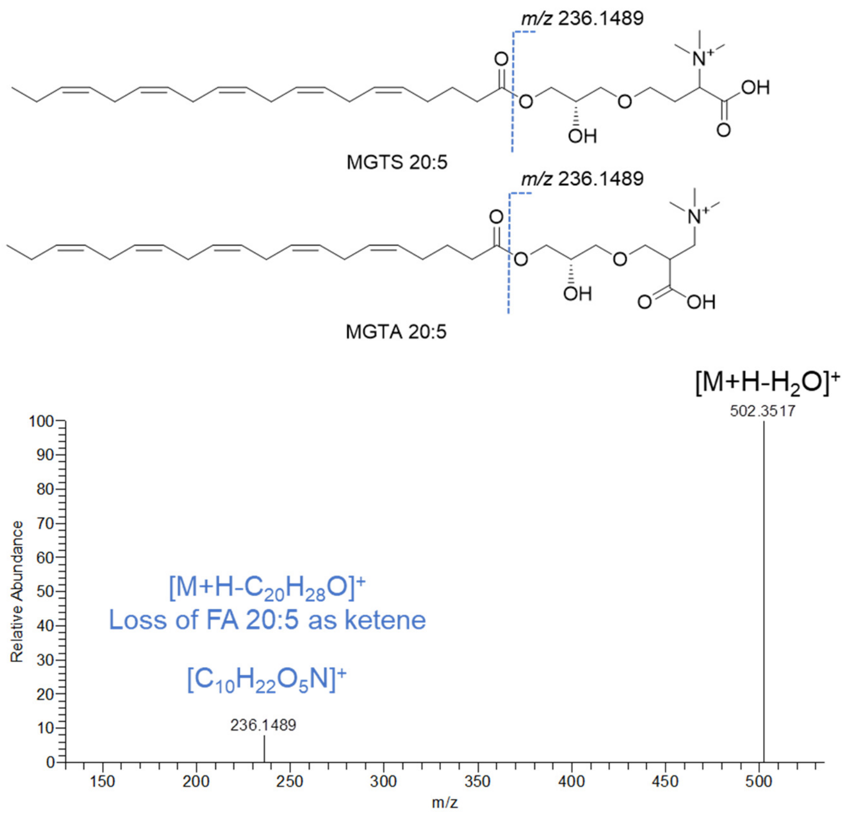

Figure S6. HR ESI-MS[2] spectra of the $[M+H]^+$ ion of MGTS/A 20:5 (*m/z* 520.3621, R_t 29.7 min) showing the diagnostic fragment for this class of lipids at *m/z* 236.14, resulting from the loss of the FA chain as a ketene. MGTS and MGTA 20:5 are structural isomers and no distinctive fragments could be observed in the tandem mass spectra of the $[M+H]^+$ adduct, to differentiate unambiguously between their structures.

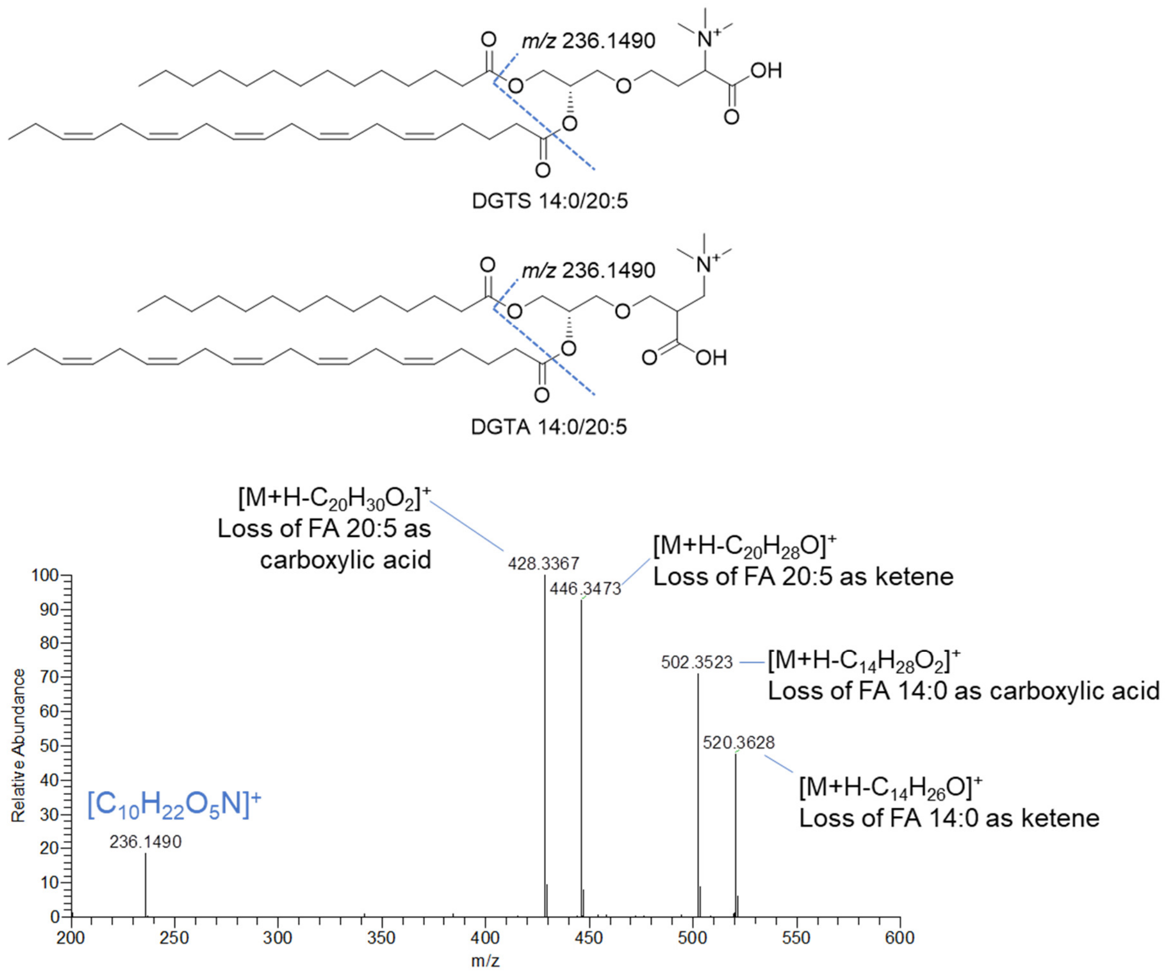

Figure S7. HR ESI-MS^2 spectra of the $[M+H]^+$ ion of DGTS/A 14:0/20:5 (*m/z* 730.5605, R_t 35.8 min) showing the diagnostic fragment for this class of lipids at *m/z* 236.14, resulting from the loss of the two FA chains as ketenes. DGTS and DGTA 14:0/20:5 are structural isomers and no distinctive fragments could be observed in the tandem mass spectra of the $[M+H]^+$ adduct, to differentiate unambiguously between their structures. Besides the fragment ion at *m/z* 236.14, the HR ESI-MS^2 spectrum of DGTS/A 14:0/20:5 displays fragment ions corresponding to the loss of each fatty acyl substituent at the *sn*-1 and *sn*-2 positions, both as ketene and carboxylic acid.

Table S1. Glycosylmonoacylglycerols identified in the bioactive fractions D and E from *Nannochloropsis granulata.*

**Glycosylmonoacylglycerols (MGMG)**					
**compound**	***R*$_t$ (min.)**	**[M+H]$^+$**	**m/z**	**MN node**	**diagnostic fragment m/z**
MGMG 20:5; O2	26.4	$C_{29}H_{47}O_{11}$	571.3103	588.3367, $[M+NH_4]^+$<br>373.2366, $[M+H-2H_2O-C_6H_{10}O_5]^+$	373.2366, from neutral losses of $2xH_2O$ and an hexosyl residue
MGMG 20:5; O	26.7	$C_{29}H_{47}O_{10}$	555.3142	375.2523, $[M+H-H_2O-C_6H_{10}O_5]^+$	375.2523, from neutral losses of H_2O and an hexosyl residue
MGMG 14:0	29.2	$C_{23}H_{45}O_9$	465.3049	303.2524, $[M+H-C_6H_{10}O_5]^+$	303.2524, from neutral loss of an hexosyl residue
MGMG 20:5	29.5	$C_{29}H_{47}O_9$	539.3202	556.3468, $[M+NH_4]^+$<br>377.2679, $[M+H-C_6H_{10}O_5]^+$	377.2684, from neutral loss of an hexosyl residue
MGMG 20:4	30.5	$C_{29}H_{49}O_9$	541.3358	563.3181, $[M+Na]^+$	379.2838, from neutral loss of an hexosyl residue
MGMG 16:0	30.7	$C_{25}H_{49}O_9$	493.3361	515.3180, $[M+Na]^+$	331.2828, from neutral loss of an hexosyl residue

Abbreviations: *MN*, molecular network. Compounds are referred to by the LIPID MAPS abbreviations (Fahy et al., 2009).

Table S2. Glycerophospholipids identified in the bioactive fractions D and E from *Nannochloropsis granulata.*

**Glycerophospholipids**					
**compound**	***R*$_t$ (min.)**	**[M+H]$^+$**	**m/z**	**MN node**	**diagnostic fragment m/z**
LPC 16:1; O2 [a]	25.2	$C_{24}H_{49}NO_9P$	526.3126	526.3126, $[M+H]^+$	184.0729, $[C_5H_{15}NO_4P]^+$ (phosphocholine)
LPC 20:5	29.3	$C_{28}H_{49}NO_7P$	542.3230	542.3230, $[M+H]^+$	184.0730, $[C_5H_{15}NO_4P]^+$ (phosphocholine)
LPC 16:1	29.5	$C_{24}H_{49}NO_7P$	494.3232	494.3232, $[M+H]^+$	184.0730, $[C_5H_{15}NO_4P]^+$ (phosphocholine)
LPE 20:4	30.3	$C_{25}H_{45}NO_7P$	502.2919	502.2919, $[M+H]^+$	361.2733, from neutral loss of phosphoethanolamine (141.0188, $C_2H_8NO_4P$)
LPC 20:4	30.3	$C_{28}H_{51}NO_7P$	544.3389	544.3389, $[M+H]^+$	184.0732, $[C_5H_{15}NO_4P]^+$ (phosphocholine)
LPT 20:4	30.6	$C_{27}H_{47}NO_9P$	560.2973	560.2973, $[M+H]^+$	361.2729, from neutral loss of phosphothreonine (199.0244, $C_4H_{10}NO_6P$)
LPC 18:1	31.0	$C_{26}H_{53}NO_7P$	522.3547	522.3547, $[M+H]^+$	184.0731, $[C_5H_{15}NO_4P]^+$ (phosphocholine)

Abbreviations: *LPC*, lyso-phosphatidylcholines; *LPE*, lyso-phosphatidylethanolamines; *LPT*, lyso-phosphatidylthreonine; *MN*, molecular network. Compounds are referred to by the LIPID MAPS abbreviations (Fahy et al., 2009).

[a] LPC with a putative hydroperoxyhexadecenoic acid as fatty acyl substituent, as revealed by a neutral loss of 34.0055 Da from the $[M+H]^+$ ion, arising from fragmentation of the hydroperoxy group.

Table S3. Fatty acids identified in the bioactive fractions D and E from *Nannochloropsis granulata.*

**Fatty acids (FA)**				
**compound**	R_t **(min.)**	$[M+H]^+$	**m/z**	**MN node**
FA 18:6; O	20.8	$C_{18}H_{27}O_3$	291.1946	273.1842, $[M+H-H_2O]^+$
FA 18:6	23.7	$C_{18}H_{25}O_2$	273.1842	255.1738, $[M+H-H_2O]^+$
FA 18:6	25.2	$C_{18}H_{25}O_2$	273.1842	255.1738, $[M+H-H_2O]^+$
FA 20:5; O2	27.7	$C_{20}H_{31}O_4$	335.2212	317.2105, $[M+H-H_2O]^+$
FA 20:5; O	28.2	$C_{20}H_{31}O_3$	319.2264	301.2157, $[M+H-H_2O]^+$<br>341.2081, $[M+Na]^+$
FA 20:5	31.4	$C_{20}H_{31}O_2$	303.2313	303.2313, $[M+H]^+$

Abbreviations: *MN*, molecular network. Compounds are referred to by the LIPID MAPS abbreviations (Fahy et al., 2009)

References

Fahy E., Subramaniam S., Murphy R. C., Nishijima M., Raetz C. R., Shimizu T., et al. (2009). Update of the LIPID MAPS Comprehensive Classification System for Lipids1. *J. Lipid Res.* 50, S9–S14. doi: 10.1194/jlr.R800095-JLR200.

Table S4: List of all cell death genes analysed, involved in apoptosis, necrosis and autophagy (some genes are involved in more than one cell death mechanism).

GENBANK	SYMBOL	DESCRIPTION	FOLD REGULATION
NM_005157	ABL1	C-abl oncogene 1, non-receptor tyrosine kinase	-1.96
NM_005163	AKT1	V-akt murine thymoma viral oncogene homolog 1	-13.27
NM_001160	APAF1	Apoptotic peptidase activating factor 1	-38.07
NM_000484	APP	Amyloid beta (A4) precursor protein	-15.53
NM_004707	ATG12	ATG12 autophagy related 12 homolog (*S. cerevisiae*)	2.69
NM_017974	ATG16L1	ATG16 autophagy related 16-like 1 (*S. cerevisiae*)	3.09
NM_022488	ATG3	ATG3 autophagy related 3 homolog (*S. cerevisiae*)	-14.54
NM_004849	ATG5	ATG5 autophagy related 5 homolog (*S. cerevisiae*)	3.02
NM_006395	ATG7	ATG7 autophagy related 7 homolog (*S. cerevisiae*)	-8.40

NM_130463	ATP6V1G2	ATPase, H+ transporting, lysosomal 13kDa, V1 subunit G2	-1.22
NM_004324	BAX	BCL2-associated X protein	-28.93
NM_000633	BCL2	B-cell CLL/lymphoma 2	-1.62
NM_004049	BCL2A1	BCL2-related protein A1	4.64
NM_138578	BCL2L1	BCL2-like 1	-1.28
NM_006538	BCL2L11	BCL2-like 11 (apoptosis facilitator)	-1.96
NM_003766	BECN1	Beclin 1, autophagy related	22.68
NM_001166	BIRC2	Baculoviral IAP repeat containing 2	-1.23
NM_001165	BIRC3	Baculoviral IAP repeat containing 3	3.60
NM_033503	BMF	Bcl2 modifying factor	-1.46
NM_017891	C1orf159	Chromosome 1 open reading frame 159	-1.21
NM_033292	CASP1	Caspase 1, apoptosis-related cysteine peptidase (interleukin 1, beta, convertase)	-1.57
NM_032982	CASP2	Caspase 2, apoptosis-related cysteine peptidase	5.99
NM_004346	CASP3	Caspase 3, apoptosis-related cysteine peptidase	-9.80
NM_032992	CASP6	Caspase 6, apoptosis-related cysteine peptidase	-1.32
NM_001227	CASP7	Caspase 7, apoptosis-related cysteine peptidase	-1.43
NM_001229	CASP9	Caspase 9, apoptosis-related cysteine peptidase	-10.74
NM_213607	CCDC103	Coiled-coil domain containing 103	1.00
NM_001250	CD40	CD40 molecule, TNF receptor superfamily member 5	-1.29
NM_000074	CD40LG	CD40 ligand	-1.22
NM_003879	CFLAR	CASP8 and FADD-like apoptosis regulator	-1.43
NM_017828	COMMD4	COMM domain containing 4	-1.32
NM_001908	CTSB	Cathepsin B	-1.78
NM_004079	CTSS	Cathepsin S	-1.43
NM_015247	CYLD	Cylindromatosis (turban tumor syndrome)	-14.38
NM_005218	DEFB1	Defensin, beta 1	-1.22
NM_005848	DENND4A	DENN/MADD domain containing 4A	-1.01
NM_004401	DFFA	DNA fragmentation factor, 45kDa, alpha polypeptide	-1.02
NM_006426	DPYSL4	Dihydropyrimidinase-like 4	-1.22
NM_015904	EIF5B	Eukaryotic translation initiation factor 5B	-1.32
NM_000125	ESR1	Estrogen receptor 1	-1.22
NM_000043	FAS	Fas (TNF receptor superfamily, member 6)	-1.42

NM_000639	FASLG	Fas ligand (TNF superfamily, member 6)	-1.29
NM_012188	FOXI1	Forkhead box I1	-1.22
NM_000152	GAA	Glucosidase, alpha; acid	9.70
NM_001924	GADD45A	Growth arrest and DNA-damage-inducible, alpha	-8.23
NM_014568	GALNT5	UDP-N-acetyl-alpha-D-galactosamine:polypeptide N-acetylgalactosaminyltransferase 5 (GalNAc-T5)	-1.22
NM_002086	GRB2	Growth factor receptor-bound protein 2	-1.01
NM_024610	HSPBAP1	HSPB (heat shock 27kDa) associated protein 1	-1.19
NM_002111	HTT	Huntingtin	1.44
NM_000619	IFNG	Interferon, gamma	-1.22
NM_000618	IGF1	Insulin-like growth factor 1 (somatomedin C)	-1.22
NM_000875	IGF1R	Insulin-like growth factor 1 receptor	-1.09
NM_000207	INS	Insulin	-1.42
NM_001145805	IRGM	Immunity-related GTPase family, M	-1.22
NM_020655	JPH3	Junctophilin 3	-1.20
NM_014592	KCNIP1	Kv channel interacting protein 1	-1.27
NM_002361	MAG	Myelin associated glycoprotein	-1.22
NM_181509	MAP1LC3A	Microtubule-associated protein 1 light chain 3 alpha	-1.40
NM_002750	MAPK8	Mitogen-activated protein kinase 8	-1.12
NM_021960	MCL1	Myeloid cell leukemia sequence 1 (BCL2-related)	3.21
NM_003998	NFKB1	Nuclear factor of kappa light polypeptide gene enhancer in B-cells 1	18.59
NM_003946	NOL3	Nucleolar protein 3 (apoptosis repressor with CARD domain)	-3.66
NM_001004467	OR10J3	Olfactory receptor, family 10, subfamily J, member 3	-1.22
NM_001618	PARP1	Poly (ADP-ribose) polymerase 1	-1.24
NM_005484	PARP2	Poly (ADP-ribose) polymerase 2	-1.29
NM_002647	PIK3C3	Phosphoinositide-3-kinase, class 3	-1.08
NM_006505	PVR	Poliovirus receptor	1.01
NM_020387	RAB25	RAB25, member RAS oncogene family	-1.25
NM_003161	RPS6KB1	Ribosomal protein S6 kinase, 70kDa, polypeptide 1	-9.55
NM_176823	S100A7A	S100 calcium binding protein A7A	-1.22
NM_000345	SNCA	Synuclein, alpha (non A4 component of amyloid precursor)	-1.21
NM_006038	SPATA2	Spermatogenesis associated 2	7.96
NM_003900	SQSTM1	Sequestosome 1	-1.14
NM_014258	SYCP2	Synaptonemal complex protein 2	-1.22
NM_018202	TMEM57	Transmembrane protein 57	-1.29
NM_000594	TNF	Tumor necrosis factor	-1.22
NM_003844	TNFRSF10A	Tumor necrosis factor receptor superfamily, member 10a	-21.16
NM_002546	TNFRSF11B	Tumor necrosis factor receptor superfamily, member 11b	9.20
NM_001065	TNFRSF1A	Tumor necrosis factor receptor superfamily, member 1A	-1.45
NM_000546	TP53	Tumor protein p53	1.10

NM_021138	TRAF2	TNF receptor-associated factor 2	-1.05
NM_017853	TXNL4B	Thioredoxin-like 4B	-1.55
NM_003565	ULK1	Unc-51-like kinase 1 (C. elegans)	11.37
NM_001167	XIAP	X-linked inhibitor of apoptosis	-1.17
